# A novel ^18^F-labelled high affinity agent for PET imaging of the translocator protein†
†Electronic supplementary information (ESI) available: Experimental procedures, spectroscopic data for all compounds synthesised, details of biological evaluation and HPLC methods, as well as NMR spectra for all key compounds. See DOI: 10.1039/c5sc01647a



**DOI:** 10.1039/c5sc01647a

**Published:** 2015-05-27

**Authors:** Adele Blair, Filip Zmuda, Gaurav Malviya, Adriana A. S. Tavares, Gilles D. Tamagnan, Anthony J. Chalmers, Deborah Dewar, Sally L. Pimlott, Andrew Sutherland

**Affiliations:** a WestCHEM , School of Chemistry , University of Glasgow , The Joseph Black Building , Glasgow G12 8QQ , UK . Email: Andrew.Sutherland@glasgow.ac.uk ; Fax: +44 (0)141 330 4888 ; Tel: +44 (0)141 330 5936; b Wolfson Whol Cancer Research Centre , Institute of Cancer Sciences , University of Glasgow , Glasgow G61 1QH , UK; c Nuclear Imaging Facility , The Beatson Institute for Cancer Research , Glasgow G61 1BD , UK; d Molecular NeuroImaging, and LLC , 60 Temple Street , New Haven , Connecticut , USA; e Institute of Neuroscience and Psychology , College of Medical , Veterinary and Life Sciences , University of Glasgow , Glasgow G12 8QQ , UK; f West of Scotland Radionuclide Dispensary , University of Glasgow and North Glasgow University Hospital NHS Trust , Glasgow G11 6NT , UK

## Abstract

The translocator protein (TSPO) is an important target for imaging focal neuroinflammation in diseases such as brain cancer, stroke and neurodegeneration, but current tracers for non-invasive imaging of TSPO have important limitations. We present the synthesis and evaluation of a novel 3-fluoromethylquinoline-2-carboxamide, AB5186, which was prepared in eight steps using a one-pot two component indium(iii)-catalysed reaction for the rapid and efficient assembly of the 4-phenylquinoline core. Biological assessment and the implementation of a physicochemical study showed AB5186 to have low nanomolar affinity for TSPO, as well as optimal plasma protein binding and membrane permeability properties. Generation of [^18^F]-AB5186 through ^18^F incorporation was achieved in good radiochemical yield and subsequent *in vitro* and *ex vivo* autoradiography revealed the ability of this compound to bind with specificity to TSPO in mouse glioblastoma xenografts. Initial positron emission tomography imaging of a glioma bearing mouse and a healthy baboon support the potential for [^18^F]-AB5186 use as a radiotracer for non-invasive TSPO imaging *in vivo*.

## Abstract 2

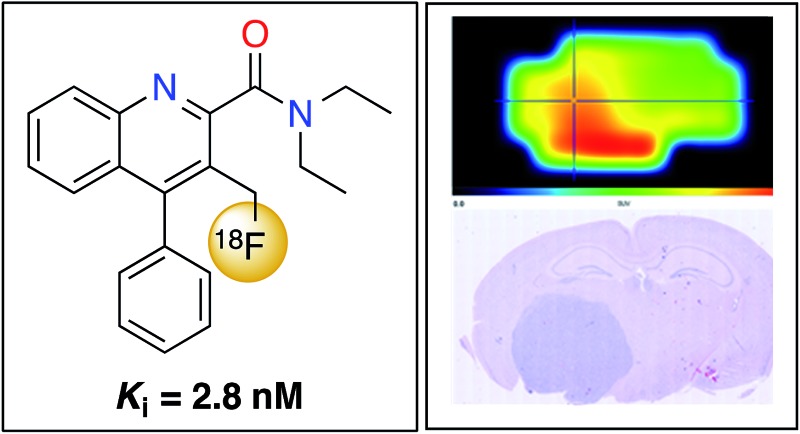
A novel ^18^F-labelled quinoline-2-carboxamide has been characterised as a novel PET imaging agent for the translocator protein.

## Introduction

Positron emission tomography (PET) is now used routinely for non-invasive imaging of human anatomy and physiology.^1^ The development of PET technology and instrumentation has allowed for the *in vivo* study of biological processes at the molecular and cellular levels.^2,3^ These studies have resulted in numerous healthcare applications such as clinical diagnosis and information on prognosis for many diseases associated with neurology, oncology and cardiology.^1,2^ PET imaging has also been used during the drug discovery process for understanding drug action and establishing dosage regimens and treatment strategies. The continued growth and increasing importance of PET imaging has driven the need for new imaging probes and chemical methods for the rapid radiolabelling of compounds. Substantial progress has been made in developing efficient transformations for the incorporation of short-lived positron-emitting isotopes,^4,5^ however a significant limitation that still exists is the discovery of new imaging agents that can bind with high affinity and specificity to key biological targets.

One such important biological target is the translocator protein (TSPO; 18 kDa), an outer mitochondrial membrane protein found in many of the major organs including lung, heart, liver, kidney and brain.^6,7^ In healthy brain tissue, TSPO is expressed at low concentrations, however in response to focal brain injury or neurodegeneration, levels of TSPO expression increase dramatically. This change has been directly linked to activation of the brain's resident immune cells, microglia,^8^ which release pro-inflammatory cytokines during infection or injury.^9^ Microglial activation and associated TSPO overexpression are therefore an indicator of the early stages of neuroinflammation associated with brain tumours^10^ and stroke-induced brain injury^11^ as well as in human neurodegenerative diseases such as Alzheimer's disease, Huntington's disease, Parkinson's disease and multiple sclerosis.^12^ For this reason, TSPO is considered an excellent target for understanding and treating diseases associated with neuroinflammation.

**Fig. 1:**
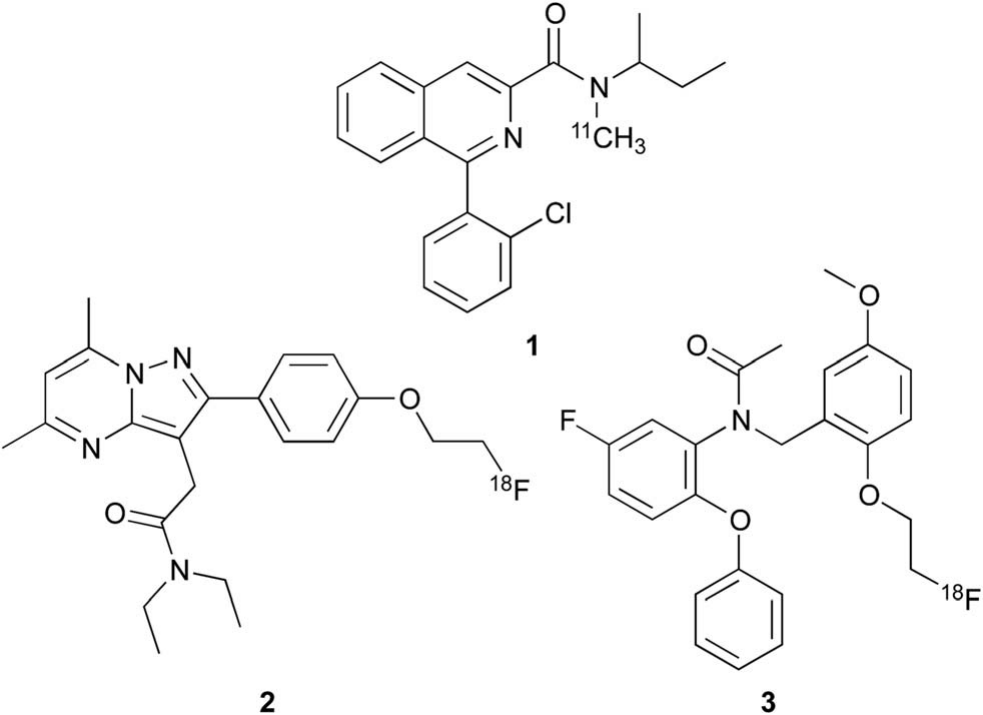
Imaging agents for TSPO; [^11^C]-PK11195 (**1**), [^18^F]-DPA714 (**2**) and [^18^F]-FEDAA1106 (**3**).

The overexpression of TSPO in active disease states has resulted in the development of many high affinity agents for *in vivo* imaging.^12,13^ The mostly widely used ligand is the isoquinoline carboxamide [^11^C]-PK11195 **1** (Fig. 1), which has been used to study patients with various disorders such as dementia, Parkinson's disease and stroke.^7,12–14^ Despite its widespread use [^11^C]-PK11195 **1** has a number of significant limitations as it has poor brain uptake and displays a low signal to noise ratio.^12,13^ In addition, labelling of this probe with the ^11^C radionuclide is challenging due to this isotope being short lived with a half-life (*t*_1/2_) of just 20.4 min. The ^18^F radionuclide has a longer *t*_1/2_ of 110 min and therefore is much more favourable and less restricting for patient imaging. Efforts have focused on the development of second generation TSPO imaging agents with improved physicochemical properties, such as [^18^F]-DPA714 **2** and [^18^F]-FEDAA1106 **3**.^12,13^ However, with the exception of PK11195, the *in vitro* binding of more recently developed TSPO PET ligands to human brain tissue is variable between different individuals such that they can be classified into high-affinity, low-affinity or mixed-affinity “binders”.^15^ This variability in the affinities of the ligands for TSPO across different individuals limits quantitative comparison of TSPO expression between subjects in PET studies.

**Fig. 2:**
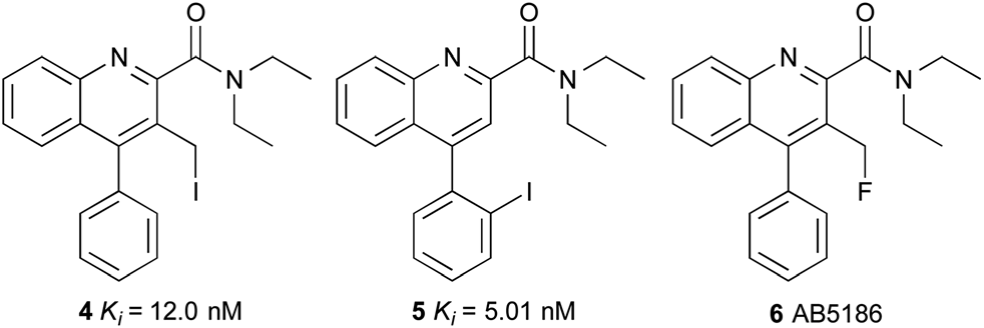
Quinoline-2-carboxamides **4**, **5** and **6**.

We therefore initiated a programme of research with the aim of developing an effective TSPO imaging agent for general use in humans.^16^ Inspired by the work of Cappelli and co-workers,^17^ we focused on quinoline-2-carboxamides that are structurally similar to PK11195 **1**. Our studies revealed the rigidity and steric factors that necessitate efficient binding of compounds to TSPO.^16^ Based on this understanding, we discovered 3-iodomethylquinoline-2-carboxamide **4** and 4-(2-iodophenyl)quinoline-2-carboxamide **5** as high affinity agents for TSPO with the potential to act as single photon emission computed tomography (SPECT) tracers (Fig. 2). Our work on the development of SPECT imaging agents for various biological targets has shown that certain negative physicochemical characteristics such as high plasma protein binding, can be due to the lipophilic nature of the iodine atom.^16,18,19^ Therefore, it was proposed that by using some of the key structural features identified from quinoline-2-carboxamides **4** and **5** in combination with a less lipophilic fluorine atom would result in an effective [^18^F]-PET imaging agent for TSPO.

We now report the synthesis, biological evaluation and physicochemical properties of novel 3-fluoromethylquinoline-2-carboxamide AB5186 **6** (Fig. 2). We describe the incorporation of the longer-lived radioactive fluorine (^18^F: *t*_1/2_ = 110 min) into AB5186 and subsequent *in vitro* and *ex vivo* autoradiography, as well as initial kinetic PET data obtained using mouse intracranial xenograft models of human glioblastoma. The brain PET kinetics and the metabolic profile of [^18^F]-AB5186 **6** obtained from a single healthy baboon are also presented.

## Results and discussion

**Scheme 1:**
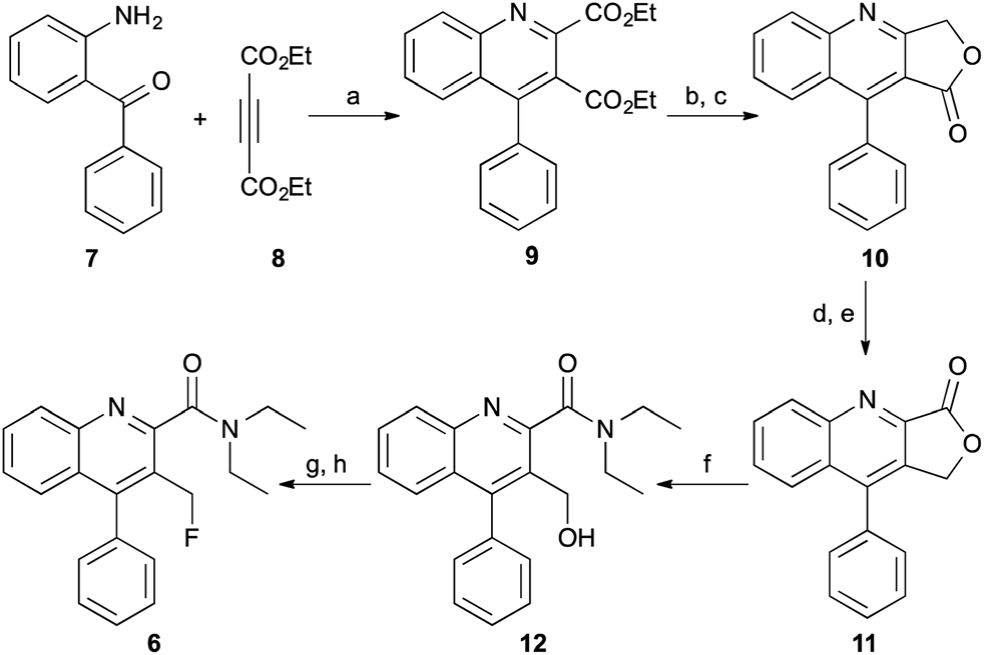
Synthesis of AB5186 **6**. *Reagents and conditions*: (a) InCl_3_, 80 °C, 3 h, 100%; (b) NaBH_4_, THF, MeOH, Δ, 15 h, 93%; (c) MnO_2_, CHCl_3_, rt, 4 h, 83%; (d) (i) LiAlH_4_, THF, 0 °C, 3 h; (ii) 10% Pd/C, MeOH, rt, 15 h, 77%; (e) MnO_2_, CHCl_3_, rt, 4 h, 92%; (f) Et_2_NH, Me_3_Al, CH_2_Cl_2_, Δ, 15 h, 61%; (g) SOCl_2_, CH_2_Cl_2_, Δ, 24 h, 100%; (h) KF, 18-crown-6, MeCN, Δ, 24 h, 60%.

The 3-fluoromethylquinoline-2-carboxamide, AB5186 **6** was prepared in eight steps as shown in Scheme 1. Previous synthetic routes to this class of compounds have used a Friedländer condensation between 2-aminobenzophenone (**7**) and ethyl 4-chloroacetoacetate,^16,17,20^ followed by an acid-mediated lactonisation which produced **10**.^21^ However, this acid-mediated lactonisation requires nine-days for completion. To circumvent this, a new approach was developed for the rapid preparation of lactone **10**. 4-Phenylquinoline **9** was prepared in quantitative yield using a one-pot two component, indium(iii)-catalysed reaction between 2-aminobenzophenone (**7**) and diethyl acetylenedicarboxylate (**8**).^22^ Reduction of diester **9** with sodium borohydride gave the corresponding 3-lactol intermediate which was subsequently oxidised to lactone **10** in high yields and more importantly, a relatively short overall reaction time. From lactone **10**, a more typical sequence of reactions were utilised to switch the lactone position from C-3 (**10**) to C-2 (**11**).^21^ Trimethylaluminium-mediated incorporation of diethylamine and consecutive formation of the 3-hydroxymethyl group gave **12** in 61% yield. Chlorination of the hydroxymethyl group followed by substitution with potassium fluoride completed the synthesis of AB5186 **6**.

Competition binding assays using rat brain homogenates to measure the displacement of [^3^H]-PK11195 revealed that AB5186 **6** has low nanomolar affinity for TSPO with a *K*_i_ value of 2.8 nM, similar to that for PK11195 **1** (Table 1). As observed with other quinoline-2-carboxamides of this class (*e.g.* **4**), the presence of the 3-fluoromethyl group likely restricts rotation of the amide, resulting in potent binding to the proposed H-bond donor pocket of TSPO.^6^

**Table 1:** Binding affinity and physicochemical values of PK11195 **1** and AB5186 **6**

Compound	*K*_i_ ^*a*^ (nM)	*P*_m_ ^*b*^	*K*_m_ ^*b*^	%PPB^*c*^
PK11195 **1**	3.1 ± 1.5	0.7	229.4	91.5
AB5186 **6**	2.8 ± 0.8	0.5	154.3	89.7

^*a*^ *K*_i_ values are the mean ± the standard deviation of three independent experiments.

^*b*^Determined using immobilised artificial membrane (IAM) column.

^*c*^Determined using human serum albumin (HSA) coated column.

The key physicochemical parameters of AB5186 **6**, permeability (*P*_m_), membrane partition coefficient (*K*_m_) and percentage of plasma protein binding (%PPB) were determined by high-performance liquid chromatography (HPLC) methods (Table 1).^23^ Using the established limits of these parameters (*P*_m_ < 0.5, *K*_m_ < 250, %PPB < 95%) for predicting *in vivo* performance of an imaging agent,^23^ AB5186 **6** was found to have optimal physicochemical properties for use as a molecular imaging tracer. A *P*_m_ of 0.5 and %PPB of 89.7% indicate that AB5186 **6** has the potential to effectively penetrate the blood brain barrier (BBB), while a *K*_m_ of 154.3 suggests that AB5186 **6** will exhibit a desirable specific to non-specific binding ratio (≥2).^23^ As AB5186 **6** contains a fluoroalkyl group, a potential alkylating agent, the stability of this compound was examined in simulated physiological conditions. Incubation of the compound in phosphate buffered saline at pH 7.4 and 37 °C showed minimal decomposition (0.03%) after 6 hours.^24^ The combination of a relatively strong C–F bond and the presence of bulky di-*ortho*-substituents impart a good level of stability.

**Scheme 2:**
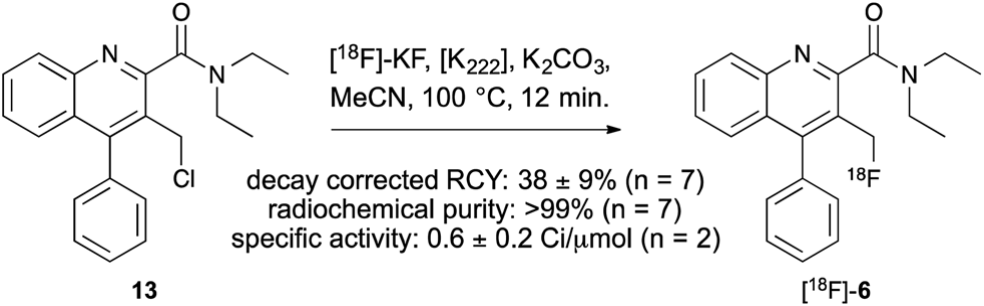
Radiosynthesis of [^18^F]-AB5186 **6**.

Encouraged by these results, a radiofluorination method for the preparation of [^18^F]-AB5186 **6** was developed which involved a halogen exchange reaction. Chloride **13**, the penultimate compound in the cold synthesis of **6** (Scheme 1) was treated with [^18^F]-potassium fluoride (607 ± 75 MBq; *n* = 7), Kryptofix® and potassium carbonate at 100 °C for 12 min (Scheme 2). After HPLC purification, [^18^F]-**6** was isolated in a 38 ± 19% decay corrected radiochemical yield (RCY) and in 118 ± 14 min (*n* = 7). The radiochemical purity of [^18^F]-AB5186 **6** was measured as >99%, with a specific activity of 0.6 ± 0.2 Ci μmol^–1^. Identification of the product was confirmed using HPLC by co-injection with a sample of unlabelled AB5186 **6**.

**Fig. 3:**
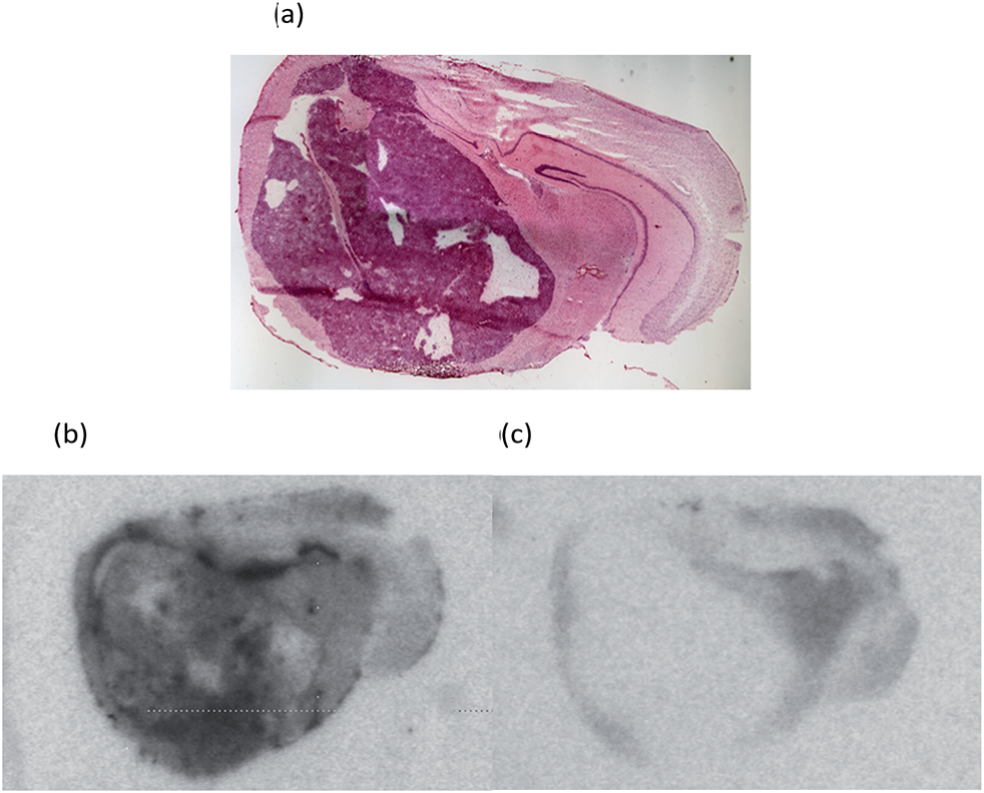
(a) H&E stained section of a CD1 nude mouse brain bearing an orthotopic G7 human glioblastoma xenograft which stains dark purple and distorts the normal brain. Representative autoradiograms showing (b) total binding of [^18^F]-AB5186 **6**, and (c) non-specific binding in the presence of unlabelled PK11195 **1**.

An initial investigation of the potential of [^18^F]-AB5186 **6** to bind to TSPO in brain involved *in vitro* autoradiography using a G7 orthotopic mouse model of human glioblastoma.^25^ Autoradiography allows the distribution of a radiolabelled compound to be visualised in intact biological tissue sections.^26^ The location of the tumour was identified by staining sections with haematoxylin and eosin, H&E, (Fig. 3a)^27^ which revealed the tumour to be large and unilateral. The *in vitro* autoradiograms showed markedly increased total binding of [^18^F]-AB5186 **6** within the tumour tissue compared to the contralateral side (Fig. 3b). This binding was displaceable in the presence of excess unlabelled PK11195 **1** (Fig. 3c) with 76% displacement in the tumour tissue compared to only 23% in the contralateral region. Displacement of [^18^F]-AB5186 **6** by the TSPO ligand PK11195 confirms the *in vitro* specificity of the tracer for TSPO in brain tissue.

**Fig. 4:**
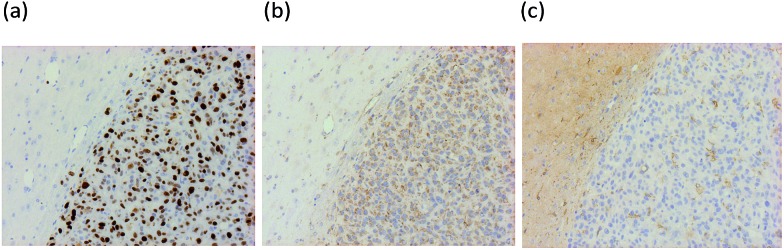
Representative immunohistochemistry images of a CD1 nude mouse brain bearing an orthotopic U87MG-luc2 glioma xenograft stained for: (a) Ki67 (cell proliferation marker), (b) TSPO, and (c) microglia (Iba1). Brown staining signifies presence of marker.

**Fig. 5:**
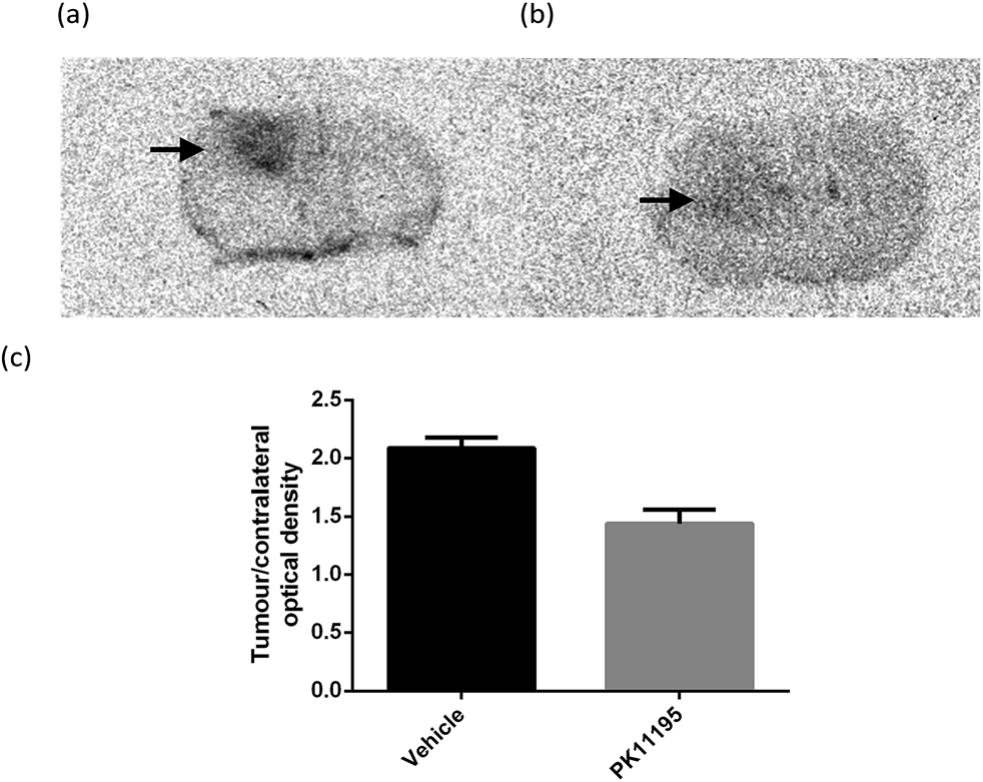
Representative coronal brain autoradiograms obtained after injecting intracranial U87MG-Luc2 glioma bearing mice with (a) vehicle or (b) 1 mg kg^–1^ PK11195 followed by [^18^F]-AB5186 10 min later. Brains were harvested for sectioning 20 min after tracer injection. Black arrows indicate tumour hotspots. (c) Ratios of tumour to contralateral tissue optical densities in the vehicle (*n* = 4) and PK11195 (*n* = 4) pre-treatment cohorts (*P* = 0.0002). Error bars represent the standard error of mean.

To investigate the behavior of [^18^F]-AB5186 **6** *in vivo*, *ex vivo* autoradiography was performed using mice bearing a U87MG-Luc2 orthotopic model of human glioblastoma. Animals were pre-treated with either vehicle (*n* = 4) or 1 mg kg^–1^ unlabelled PK11195 (*n* = 4) administered intravenously 10 min prior to [^18^F]-AB5186 **6** intravenous injection. Animals were killed and brains were removed 20 min after the radiotracer was given and frozen. Cryostat sections were then used to generate autoradiograms. Histological evaluation of the intracranial U87MG-Luc2 mouse model showed a large number of proliferating cells (Fig. 4a), TSPO expression (Fig. 4b) and microglia (Fig. 4c) within the main tumour mass. The non-tumour bearing contralateral side of the brain lacked proliferating cells and minimal TSPO expression and immunolabelled microglia were visible (data not shown). Under normal circumstances microglia express low levels of TSPO in the brain. However, in response to injury microglia become activated and TSPO expression is greatly increased.^8^ However, in this case it is presumed that the cancer cells contribute to the majority of the TSPO overexpression.^28^ The total binding of [^18^F]-AB5186 **6** was substantially higher in the tumour tissue compared to the contralateral region (Fig. 5a and c). The binding was significantly reduced (*P* = 0.0002) in animals treated with unlabelled PK11195 (Fig. 5b and c) confirming the *in vivo* specificity of [^18^F]-AB5186 **6** for TSPO.

**Fig. 6:**
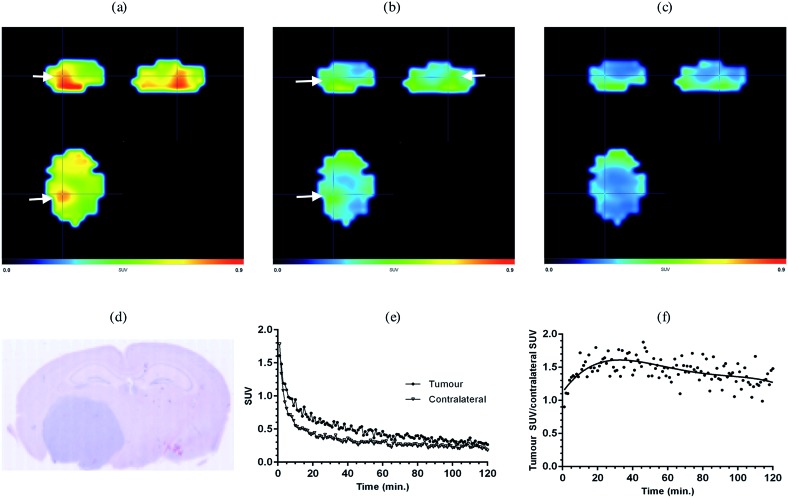
Averaged PET images of a U87MG-Luc2 glioma bearing mouse brain following [^18^F]-AB5186 injection: (a) 0–40 min averaged, (b) 41–80 min averaged, and (c) 81–120 min averaged; 120 min dynamic scan. White arrows indicate tumour hotspots. The brain was defined using the skull following manual co-registration of the CT image with the PET image. (d) H&E staining showing location of the tumour which stains dark blue. (e) Time–activity curves of the tracer in the tumour and contralateral brain regions obtained from manually defined VOIs. (f) Tracer kinetics expressed as a ratio of the tumour to contralateral SUVs.

In order to investigate the potential of [^18^F]-AB5186 **6** as a PET tracer for TSPO a dynamic PET scan was performed using the Albira imaging system (Carestream Molecular Imaging, USA) in a single mouse bearing an intracranial U87MG-Luc2 tumour for 120 min after intravenous administration of the tracer. Postmortem histological examination of the brain collected at the end of the scan was conducted to visualise the anatomical location of the tumour. Following reconstruction, the entire dynamic PET image was manually co-registered to key anatomical structures of the CT (*i.e.* the spine, the front limbs and the eye sockets). The skull was then used to define the brain region and the PET signal outside of this region was masked which allowed for greater clarity when identifying the tumour hotspot. Binding of [^18^F]-AB5186 **6** to the tumour was clearly visible in the PET scan (Fig. 6a–c) whose location correlated to that revealed by histology (Fig. 6d). Time–activity curve analysis of the PET data revealed greater amounts of [^18^F]-AB5186 **6** in the tumour *versus* the contralateral region during the early portion of the scan (Fig. 6e and f). These data support the ability of [^18^F]-AB5186 **6** to image TSPO *in vivo* under pathological conditions.

**Fig. 7:**
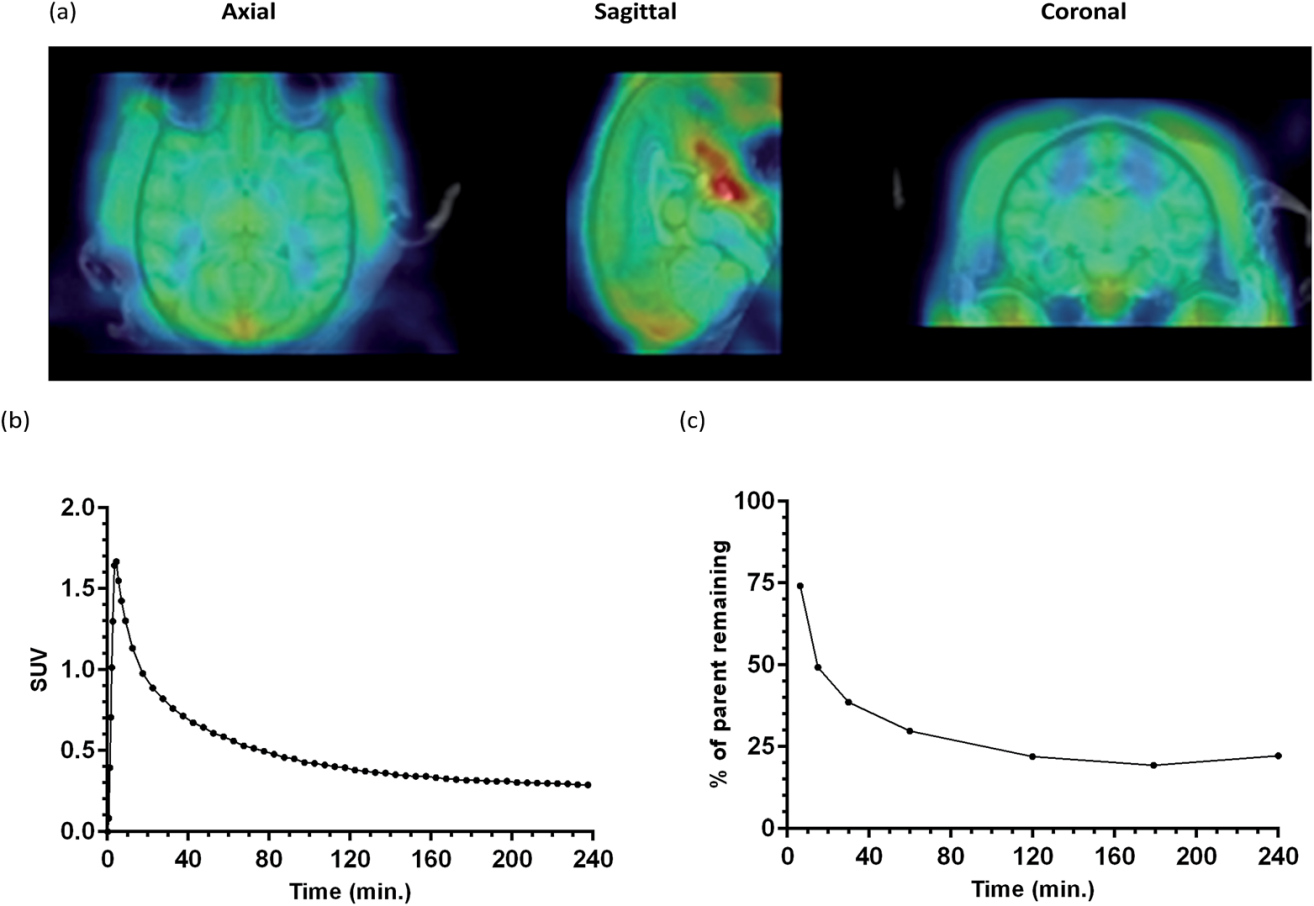
(a) MRI and averaged PET image (0–60 min; 240 min dynamic scan) of healthy baboon brain following [^18^F]-AB5186 **6** injection. (b) Whole brain time activity curve for the tracer in the baboon brain. (c) Parent fraction profile of [^18^F]-AB5186 **6** over time following bolus injection into baboon.

In order to show that [^18^F]-AB5186 **6** can penetrate into healthy brain tissue, a 240 min baseline dynamic PET scan was performed of the head of a healthy baboon. Following PET reconstruction and co-registration to an MRI (Fig. 7a), a time–activity curve for the tracer in the whole brain was established (Fig. 7b). The peak standardised uptake values (SUV) in the baboon brain was 1.67 suggesting that [^18^F]-AB5186 **6** can effectively cross the intact BBB. Metabolic analysis of arterial blood samples taken from the baboon during PET imaging (Fig. 7c) revealed moderate metabolism of [^18^F]-AB5186 **6**, with 29.8% of the parent still intact 60 minutes post-injection (Fig. 7c).

## Conclusions

We have developed a novel 3-fluoromethylquinoline-2-carboxamide, AB5186 **6**, which has low nanomolar affinity for TSPO and optimal physicochemical properties. Radiofluorination methodology was developed allowing efficient access to the radiolabelled version of this compound ([^18^F]-AB5186 **6**). *In vitro* and *ex vivo* autoradiography studies in mouse models of human glioblastoma, showed specific binding of [^18^F]-AB5186 to TSPO in tumour tissue. In addition, [^18^F]-AB5186 **6** exhibited the ability to image TSPO in an intracranial glioma bearing mouse and to penetrate the intact BBB in a non-human primate. Taken together these findings support the potential for further development of [^18^F]-AB5186 as a PET imaging agent for TSPO in the brain.

Recently, Pike and co-workers reported low sensitivity of [^11^C]-labelled azaisosteres of [^11^C]-PK11195 **1** to TSPO single nucleotide polymorphism.^29^ We hypothesise that because of the restricted rotation of the structure, [^18^F]-AB5186 **6** will also possess low sensitivity to this polymorphism. These properties in combination with the longer-lived ^18^F radioisotope (^18^F: *t*_1/2_ = 110 min *versus* ^11^C: *t*_1/2_ = 20.4 min) should allow the general application of [^18^F]-AB5186 **6** in preclinical and clinical imaging studies of brain pathologies associated with TSPO overexpression. Further evaluation of the binding profile of [^18^F]-AB5186 **6** in human tissue is currently underway in our laboratories.
