# Supplementary material for: A novel 18F-labelled high affinity agent for PET imaging of the translocator protein
Source: Chem Sci. 2015 May 27;6(8):4772–7. doi: 10.1039/c5sc01647a (PMC5667507; doi:10.1039/c5sc01647a)
Supplement: Supplementary file 1 [file SC-006-C5SC01647A-s001.pdf]

Electronic Supplementary Material (ESI) for Chemical Science.
This journal is © The Royal Society of Chemistry 2015

Supporting Information for:

A novel ^{18}F-labelled high affinity agent for PET imaging of the translocator protein

Adele Blair,[a] Filip Zmuda,[a,b] Gaurav Malviya,[c] Adriana A. S. Tavares,[d] Gilles D. Tamagnan,[d] Anthony J. Chalmers,[b] Deborah Dewar,[e] Sally L. Pimlott[f] and Andrew Sutherland[a]*

[a]WestCHEM, School of Chemistry, The Joseph Black Building, University of Glasgow, Glasgow G12 8QQ, UK. [b]Wolfson Whol Cancer Research Centre, Institute of Cancer Sciences, University of Glasgow, Glasgow G61 1QH, UK. [c]Nuclear Imaging Facility, The Beatson Institute for Cancer Research, Glasgow G61 1BD, UK. [d]Molecular NeuroImaging, LLC, 60 Temple Street, New Haven, Connecticut, USA. [e]Institute of Neuroscience and Psychology, College of Medical, Veterinary and Life Sciences, University of Glasgow, Glasgow G12 8QQ, UK. [f]West of Scotland Radionuclide Dispensary, University of Glasgow and North Glasgow University Hospital NHS Trust, Glasgow G11 6NT, UK.

Email: Andrew.Sutherland@glasgow.ac.uk; Fax: 0141 330 4888; Tel: 0141 330 5936.

Table of Contents

1. General Experimental S2
2. Experimental Procedures and Spectroscopic Data for all Compounds S2–S8
3. HPLC Methodology S8–S9
4. Radioligand Binding Methodology S10–S11
5. Binding Curves S11
6. Stability Testing S12
7. Radiochemistry Methodology S12–S13
8. Radio-HPLC chromatogram of [^{18}F]-AB5186 **6** after purification S13
9. Mouse Glioma Models S13
10. *In Vitro* Autoradiography Methodology S13–S14
11. Immunohistochemistry Methodology S14
12. *Ex Vivo* Autoradiography Methodology S14–S15
13. Small Animal PET Methodology S15–S16
14. Primate PET Methodology S16–S17
15. Time Activity Curves of [^{18}F]-AB5186 **6** in Baboon S18
16. References S18
17. ^{1}H and ^{13}C NMR Spectra for New Compounds S19–S26

1. General Experimental

All reagents and starting materials were obtained from commercial sources and used as received. All dry solvents were purified using a PureSolv 500 MD solvent purification system. All reactions were performed under argon unless otherwise stated. Brine is defined as a saturated solution of aqueous sodium chloride. Flash column chromatography was carried out using Fisher Matrix silica 60. Macherey–Nagel aluminium–backed plates pre–coated with silica gel 60 (UV_{254}) were used for thin layer chromatography and were visualized using UV light. ^{1}H NMR and ^{13}C NMR spectra were recorded on a Bruker DPX 400 spectrometer or Bruker 500 spectrometer with chemical shift values in ppm relative to tetramethylsilane (δ_H 0.00 and δ_C 0.0) or residual chloroform (δ_H 7.26 and δ_C 77.2) as the standard. ^{1}H and ^{13}C assignments are based on two-dimensional COSY and DEPT experiments, respectively. Infrared spectra were recorded on a JASCO FTIR 410 spectrometer. Mass spectra were recorded using electron impact, chemical ionisation or fast atom bombardment techniques. HRMS spectra were recorded using a dual-focusing magnetic analyser mass spectrometer. Melting points were determined on a Gallenkamp melting point apparatus. All experiments with animals were approved by Animal Welfare and Ethical Review Board (University of Glasgow) and carried out in accordance with the Animals (Scientific Procedures) Act 1986 and guidelines of the Committee of the National Cancer Research Institute.

2. Experimental Procedures and Spectroscopic Data for All Compounds

Diethyl 4-phenylquinoline-2,3-dicarboxylate (9)[1]

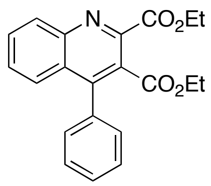

Indium(III) chloride (0.68 g, 3.07 mmol) was added to 2-aminobenzophenone (**7**) (5.00 g, 15.3 mmol) and diethyl acetylenedicarboxylate (**8**) (2.95 mL, 18.4 mmol), and the neat mixture stirred at 80 °C for 3 h. On cooling to ambient temperature, water (50 mL) was added and the mixture extracted using ethyl acetate (150 mL). The organic layer was then washed with water (100 mL) and brine (100 mL), dried ($MgSO_4$), filtered and concentrated *in vacuo*. Purification by silica column chromatography (ethyl acetate/hexane, 15:85) afforded diethyl 4-phenylquinoline-2,3-dicarboxylate (**9**) (5.36 g, 100%) as a yellow solid. Mp 89–91 °C (lit.,[1] mp 95–96 °C); ν_{max}/cm^{-1} (neat) 2984 (CH), 1741 (CO), 1722 (CO), 1560 (C=C), 1441, 1376, 1247, 1202, 1048, 768; δ_H (400 MHz, $CDCl_3$) 0.99 (3H, t, *J* 7.2 Hz, OCH_2CH_3), 1.47 (3H, t, *J* 7.2 Hz, OCH_2CH_3), 4.09 (2H, q, *J*

7.2 Hz, OCH_2CH$_3$), 4.54 (2H, q, *J* 7.2 Hz, OCH_2CH$_3$), 7.34–7.40 (2H, m, 2 × ArH), 7.47–7.53 (3H, m, 3 × ArH), 7.57 (1H, ddd, *J* 8.4, 6.4, 1.6 Hz, ArH), 7.63 (1H, ddd, *J* 8.4, 1.6, 0.6 Hz, ArH), 7.81 (1H, ddd, *J* 8.4, 6.4, 1.6 Hz, ArH), 8.38 (1H, ddd, *J* 8.4, 1.6, 0.6 Hz, ArH); δ_C (101 MHz, $CDCl_3$) 13.6 (CH_3), 14.2 (CH_3), 61.6 (CH_2), 62.6 (CH_2), 126.6 (CH), 127.1 (C), 127.5 (C), 128.2 (2 × CH), 128.7 (CH), 129.0 (CH), 129.4 (2 × CH), 130.7 (CH), 130.9 (CH), 134.8 (C), 145.9 (C), 147.1 (C), 148.0 (C), 165.3 (C), 167.2 (C); *m/z* (EI) 349.1311 (M^+. $C_{21}H_{19}NO_4$ requires 349.1314), 305 (20%), 276 (55), 205 (100), 204 (82), 165 (12), 84 (5).

9-Phenyl-1*H*,3*H*-furo[3,4-*b*]quinolin-1-ol

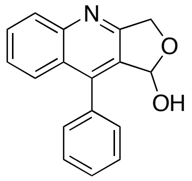

A solution of ethyl 4-phenylquinoline-2,3-dicarboxylate (**9**) (4.72 g, 13.5 mmol) in tetrahydrofuran (50 mL) was heated under reflux with vigorous stirring. To this was added dropwise, a solution of sodium borohydride (5.11 g, 135 mmol) in methanol (50 mL), and the resultant reaction mixture stirred under reflux for 15 h. After cooling to ambient temperature, excess sodium borohydride was quenched by the dropwise addition of a saturated solution of ammonium chloride (~ 50 mL). The crude reaction mixture was extracted using dichloromethane (3 × 50 mL) and washed with water (2 × 50 mL), dried ($MgSO_4$), filtered and concentrated *in vacuo*. Trituration with diethyl ether afforded 9-phenyl-1*H*,3*H*-furo[3,4-b]quinolin-1-ol as a pale yellow solid (3.29 g, 93%). Mp 208–210 °C; ν_{max}/cm^{-1} (neat) 3327 (OH), 2927 (CH), 1727, 1538, 1486, 1443, 1213, 1026, 749; δ_H (400 MHz, DMSO-d_6) 4.87 (1H, dd, *J* 15.7, 0.8 Hz, CH*H*O), 4.98 (1H, d, *J* 15.7 Hz, C*H*HO), 5.02 (1H, br s, C*H*OH), 6.87–6.94 (2H, m, 2 × ArH), 7.04 (1H, d, *J* 6.8 Hz, ArH), 7.10–7.17 (2H, m, 2 × ArH), 7.18–7.28 (4H, m, 4 × ArH), 10.0 (1H, br s, CHO*H*); δ_C (101 MHz, DMSO-d_6) 65.0 (CH_2), 95.2 (C), 116.2 (CH), 123.1 (CH), 124.3 (C), 126.2 (CH), 127.5 (CH), 127.6 (2 × CH), 128.3 (2 × CH), 130.8 (CH), 136.1 (C), 146.8 (C), 158.5 (C), 172.0 (C); *m/z* (ESI) 286.0830 (MNa^+. $C_{17}H_{13}NNaO_2$ requires 286.0838).

9-Phenylfuro[3,4-*b*]quinolin-1(3*H*)-one (10)[2]

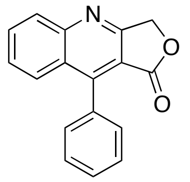

To a solution of 9-phenyl-1*H*,3*H*-furo[3,4-*b*]quinolin-1-ol (3.29 g, 12.5 mmol) in chloroform (150 mL) was added activated manganese(IV) oxide (32.6 g, 374 mmol) in one portion. The resultant suspension was stirred vigorously at ambient temperature for 4 h and then filtered through Celite®. After washing with chloroform (200 mL), the solvent was removed *in vacuo* to afford 9-phenylfuro[3,4-*b*]quinolin-1(3*H*)-one (**10**) as a white solid (2.72 g, 83%), which was used without further purification. Mp 201–203 °C (lit.,[2] mp 203–204 °C); ν_{max}/cm^{-1} (neat) 2957 (CH), 2843, 1771 (CO), 1603 (C=C), 1449, 1219, 1119, 1049, 1028, 889; δ_H (500 MHz, $CDCl_3$) 5.45 (2H, s, OCH_2), 7.43–7.49 (2H, m, 2 × ArH), 7.57–7.61 (4H, m, 4 × ArH), 7.89–7.93 (2H, m, 2 × ArH), 8.19–8.22 (1H, m, ArH); δ_C (126 MHz, $CDCl_3$) 69.6 (CH_2), 113.5 (C), 127.1 (C), 127.4 (CH), 128.1 (CH), 128.3 (2 × CH), 129.4 (CH), 129.5 (CH), 129.8 (2 × CH), 131.6 (C), 132.6 (CH), 151.3 (C), 151.5 (C), 163.6 (C), 167.9 (C); *m/z* (EI) 261.0794 (M^+. $C_{17}H_{11}NO_2$ requires 261.0790), 232 (56%), 204 (42), 203 (32), 176 (21), 151 (9), 131 (8), 91 (10).

2,3-Bis(hydroxymethyl)-4-phenylquinoline[2]

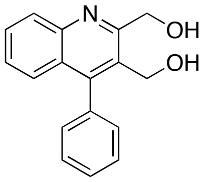

A suspension of lithium aluminium hydride (0.58 g, 15.3 mmol) in tetrahydrofuran (25 mL) was cooled to 0 °C, and to this was added dropwise, a solution of 9-phenylfuro[3,4-*b*]quinolin-1(3*H*)-one (**10**) (1.00 g, 3.83 mmol) in tetrahydrofuran (25 mL). The temperature of the reaction mixture was maintained at 0 °C and stirred vigorously for 3 h. After gradually warming to ambient temperature, the mixture was quenched by the dropwise addition of a 1 M hydrochloric acid solution, and then extracted using ethyl acetate (3 × 25 mL). The organic layer was dried ($MgSO_4$), filtered and concentrated *in vacuo* to afford a colourless residue which was dissolved in methanol (50 mL). 10% Palladium on carbon (1.00 g) was added to the reaction flask and the resultant suspension stirred at ambient temperature for 15 h. The suspension was then filtered through a pad of Celite®, washed with methanol and concentrated *in vacuo*. Trituration with diethyl ether afforded

2,3-bis(hydroxymethyl)-4-phenylquinoline as a white solid (0.777 g, 77%). Mp 171–173 °C (lit.,[2] mp 175–177 °C); ν_{max}/cm^{-1} (neat) 3422 (OH), 3074, 2991 (CH), 1571, 1490, 1442, 1231, 1022, 1006; δ_H (400 MHz, CD_3OD) 4.59 (2H, s, C*H*$_2$OH), 5.11 (2H, s, C*H*$_2$OH), 7.34–7.38 (2H, m, 2 × ArH), 7.41 (1H, dd, *J* 8.4, 1.2 Hz, ArH), 7.44–7.50 (1H, m, ArH), 7.51–7.60 (3H, m, 3 × ArH), 7.73 (1H, ddd, *J* 8.4, 6.4, 1.2 Hz, ArH), 8.11 (1H, d, *J* 8.4 Hz, ArH); δ_C (101 MHz, CD_3OD) 59.1 (CH_2), 64.6 (CH_2), 127.8 (CH), 127.9 (CH), 128.6 (C), 129.4 (CH), 129.5 (CH), 129.5 (2 × CH), 130.0 (C), 130.8 (CH), 130.8 (2 × CH), 137.2 (C), 147.4 (C), 150.4 (C), 160.9 (C); *m/z* (CI) 266.1186 (MH^+. $C_{17}H_{16}NO_2$ requires 266.1181), 264 (50%), 248 (42), 218 (21), 206 (4), 151 (3).

9-Phenylfuro[3,4-*b*]quinolin-3(1*H*)-one (11)[2]

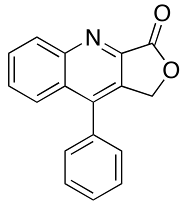

To a solution of 2,3-bis(hydroxymethyl)-4-phenylquinoline (0.78 g, 2.93 mmol) in chloroform (50 mL) was added activated manganese(IV) oxide (7.64 g, 87.9 mmol) in one portion. The resultant suspension was stirred vigorously at ambient temperature for 4 h and then filtered through Celite®. After washing with chloroform (100 mL), the solvent was removed *in vacuo* to afford 9-phenylfuro[3,4-*b*]quinolin-3(1*H*)-one (**11**) as a white solid (0.70 g, 92%), which was used without further purification. Mp 188–190 °C (lit.,[2] mp 190–192 °C); ν_{max}/cm^{-1} (neat) 3005 (CH), 1775 (CO), 1582, 1414, 1371, 1152, 1121, 1055, 1007; δ_H (400 MHz, $CDCl_3$) 5.41 (2H, s, OCH_2), 7.45 (2H, dd, *J* 8.0, 2.0 Hz, 2 × ArH), 7.57–7.70 (4H, m, 4 × ArH), 7.84–7.90 (1H, m, ArH), 7.92 (1H, d, *J* 8.4 Hz, ArH), 8.46 (1H, d, *J* 8.4 Hz, ArH); δ_C (101 MHz, $CDCl_3$) 67.8 (CH_2), 125.7 (CH), 127.9 (C), 128.9 (2 × CH), 129.3 (2 × CH), 129.4 (CH), 129.6 (CH), 130.7 (CH), 131.5 (CH), 132.3 (C), 133.6 (C), 143.9 (C), 144.4 (C), 150.7 (C), 168.7 (C); *m/z* (ESI) 284.0677 (MNa^+. $C_{17}H_{11}NNaO_2$ requires 284.0682).

3-(Hydroxymethyl)-4-phenylquinoline-2-*N*-diethylcarboxamide (12)

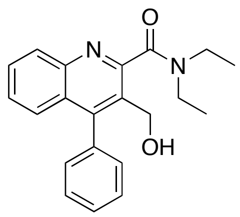

To a solution of diethylamine (0.24 mL, 2.30 mmol) in dichloromethane (20 mL) was added trimethylaluminium solution (2.0 M in toluene) (1.15 mL, 2.30 mmol) dropwise, and the mixture stirred at ambient temperature for 0.3 h. A solution of 9-phenylfuro[3,4-*b*]quinolin-3(1*H*)-one (**11**) (0.30 g, 1.15 mmol) in dichloromethane (20 mL) was then added dropwise and the resultant reaction mixture stirred under reflux for 15 h. On cooling to ambient temperature, the reaction was quenched by the careful addition of a 1 M hydrochloric acid solution (25 mL), and the crude mixture extracted using dichloromethane (2 × 20 mL). The organic layer was dried ($MgSO_4$), filtered and concentrated *in vacuo*. Purification by silica column chromatography (ethyl acetate/petroleum ether, 1:3) gave 3-(hydroxymethyl)-4-phenylquinoline-2-*N*-diethylcarboxamide (**12**) as a pale yellow solid (0.24 g, 61%). Mp 117–119 °C; ν_{max}/cm^{-1} (neat) 3408 (OH), 2972, 2932 (CH), 1613 (CO), 1483, 1443, 1267, 1216, 1069; δ_H (400 MHz, $CDCl_3$) 1.27 (3H, t, *J* 7.2 Hz, NCH_2CH_3), 1.36 (3H, t, *J* 7.2 Hz, NCH_2CH_3), 3.42 (2H, q, *J* 7.2 Hz, NCH_2CH_3), 3.68 (2H, q, *J* 7.2 Hz, NCH_2CH_3), 4.12 (1H, t, *J* 6.4 Hz, CH_2OH), 4.39 (2H, d, *J* 6.4 Hz, CH_2OH), 7.41–7.58 (7H, m, 7 × ArH), 7.72 (1H, ddd, *J* 8.4, 6.8, 1.6 Hz, ArH), 8.12 (1H, ddd, *J* 8.4, 1.2, 0.8 Hz, ArH); δ_C (101 MHz, $CDCl_3$) 12.9 (CH_3), 14.2 (CH_3), 40.3 (CH_2), 43.7 (CH_2), 59.7 (CH_2), 127.0 (CH), 127.4 (CH), 127.6 (C), 128.4 (3 × CH), 129.4 (C), 129.6 (CH), 129.7 (CH), 129.9 (2 × CH), 135.2 (C), 146.1 (C), 149.3 (C), 155.1 (C), 169.8 (C); *m/z* (ESI) 357.1576 (MNa^+. $C_{21}H_{22}N_2NaO_2$ requires 357.1573); Anal. Calcd for $C_{21}H_{22}N_2O_2$: C, 75.42; H, 6.63; N, 8.38. Found: C, 75.03; H, 6.57; N, 8.23.

3-(Chloromethyl)-4-phenylquinoline-2-*N*-diethylcarboxamide (13)

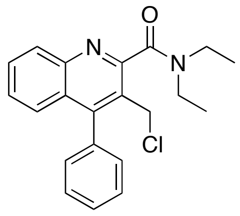

To a solution of 3-(hydroxymethyl)-4-phenylquinoline-2-*N*-diethylcarboxamide (**12**) (0.10 g, 0.30 mmol) in dichloromethane (10 mL) was added thionyl chloride (0.65 mL, 8.97 mmol) and the

reaction mixture stirred under reflux for 24 h. On cooling to ambient temperature, the mixture was concentrated *in vacuo* and azeotroped with toluene (3 × 10 mL) to remove excess thionyl chloride. Purification by silica column chromatography (diethyl ether/petroleum ether, 1:1) gave 3-(chloromethyl)-4-phenylquinoline-2-*N*-diethylcarboxamide (**13**) as a colourless oil (0.11 g, 100%). ν_{max}/cm^{-1} (neat) 2981 (CH), 2936, 1630 (CO), 1560 (C=C), 1482, 1443, 1270, 1218, 1127, 1067, 728; δ_H (500 MHz, $CDCl_3$) 1.30 (3H, t, *J* 7.0 Hz, NCH_2CH_3), 1.35 (3H, t, *J* 7.5 Hz, NCH_2CH_3), 3.28 (2H, q, *J* 7.0 Hz, NCH_2CH_3), 3.68 (2H, q, *J* 7.5 Hz, NCH_2CH_3), 4.70 (2H, s, CH_2Cl), 7.36–7.40 (2H, m, 2 × ArH), 7.41–7.49 (2H, m, 2 × ArH), 7.51–7.59 (3H, m, 3 × ArH), 7.73 (1H, ddd, *J* 8.5, 6.5, 1.5 Hz, ArH), 8.13 (1H, d, *J* 8.5 Hz, ArH); δ_C (126 MHz, $CDCl_3$) 12.3 (CH_3), 13.5 (CH_3), 39.2 (CH_2), 40.3 (CH_2), 43.6 (CH_2), 125.9 (C), 126.9 (CH), 127.3 (C), 127.4 (CH), 128.6 (2 × CH), 128.7 (CH), 129.4 (2 × CH), 129.6 (CH), 130.2 (CH), 134.9 (C), 146.4 (C), 149.4 (C), 155.6 (C), 168.1 (C); *m/z* (ESI) 375.1218 (MNa^+. $C_{21}H_{21}{}^{35}ClN_2NaO$ requires 375.1235).

3-(Fluoromethyl)-4-phenylquinoline-2-*N*-diethylcarboxamide (6)

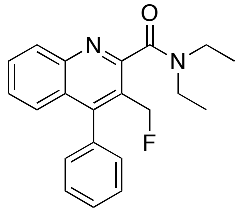

To a solution of 18-crown-6 (0.04 g, 0.14 mmol) in acetonitrile (2.5 mL) was added potassium fluoride (0.04 g, 0.71 mmol) and the resultant suspension stirred at ambient temperature for 0.5 h. A solution of 3-(chloromethyl)-4-phenylquinoline-2-*N*-diethylcarboxamide (**13**) (0.05 g, 0.14 mmol) in acetonitrile (2.5 mL) was then added dropwise to the reaction mixture and stirred under reflux for 24 h. On cooling to ambient temperature, water (10 mL) was added to the mixture and then extracted using dichloromethane (3 × 15 mL). The organic layer was dried ($MgSO_4$), filtered and concentrated *in vacuo*. Purification by silica column chromatography (diethyl ether/petroleum ether, 1:1), followed by washing with acetonitrile gave 3-(fluoromethyl)-4-phenylquinoline-2-*N*-diethylcarboxamide (**6**) as a white solid (0.03 g, 60%). ν_{max}/cm^{-1} (neat) 2976, 2936 (CH), 1634 (CO), 1560 (C=C), 1481, 1269, 1213, 1069, 970; δ_H (400 MHz, $CDCl_3$) 1.22 (3H, t, *J* 7.2 Hz, NCH_2CH_3), 1.34 (3H, t, *J* 7.2 Hz, NCH_2CH_3), 3.28 (2H, q, *J* 7.2 Hz, NCH_2CH_3), 3.67 (2H, q, *J* 7.2 Hz, NCH_2CH_3), 5.38 (2H, d, *J* 47.8 Hz, CH_2F), 7.32–7.38 (2H, m, 2 × ArH), 7.46–7.57 (5H, m, 5 × ArH), 7.73–7.78 (1H, m, ArH), 8.16 (1H, d, *J* 8.4 Hz, ArH); δ_C (101 MHz, $CDCl_3$) 12.6 (CH_3), 13.7 (CH_3), 39.4 (CH_2), 43.2 (CH_2), 79.2 (CH_2F, d, $J_{C\text{-}F}$ 162.2), 123.4 (C, d, $J_{C\text{-}C\text{-}F}$ 15.1 Hz), 127.0 (CH), 127.1 (C, d, $J_{C\text{-}C\text{-}C\text{-}C\text{-}F}$ 1.8 Hz), 127.4 (CH), 128.5 (2 × CH), 128.7 (CH), 129.6 (CH), 129.7 (2 ×

CH), 130.5 (CH, d, $J_{C\text{-}C\text{-}C\text{-}C\text{-}C\text{-}F}$ 1.5 Hz), 134.8 (C), 147.1 (C, d, $J_{C\text{-}C\text{-}C\text{-}F}$ 2.9 Hz), 150.7 (C, d, $J_{C\text{-}C\text{-}C\text{-}F}$ 4.9 Hz), 155.9 (C, d, $J_{C\text{-}C\text{-}C\text{-}C\text{-}F}$ 1.7 Hz), 168.2 (C); *m/z* (ESI) 359.1520 (MNa^+. $C_{21}H_{21}FN_2NaO$ requires 359.1530).

3. HPLC Methodology[3]

All physicochemical analyses were performed using a Dionex Ultimate 3000 series, and data acquisition and processing performed using Chromeleon 6.8 Chromatography software. Standard and test compounds were dissolved in 1:1 organic/aqueous phases, and prepared to a concentration of 0.5 mg/mL. The HPLC system was set to 25 °C, and UV detection achieved using a diode array detector (190–800 nm). Analysis was performed using 5 μL sample injections.

Immobilised Artificial Membrane (IAM) chromatography for determination of membrane permeability (P_m) and membrane partition coefficient (K_m)

P_m and K_m values were determined using previously developed methodology on a Registech IAM.PC.DD2 (15 cm × 4.6 mm) column. Acetonitrile and 0.01 mM phosphate buffered saline at pH 7.4 was used as the mobile phase, with a flow rate of 1.0 mL/min. The retention time of each compound was measured under an isocratic mobile phase with the percentage of acetonitrile ranging from 30–40%. The retention time of citric acid as an unretained compound under an isocratic mobile phase of 100% phosphate buffered saline was used for system corrections. The following equations were used to calculate P_m and K_m of the compounds of interest using Excel 2008 Software.

$$k_{IAM} = \frac{(t_r - t_0)}{t_0}$$

where k_{IAM} = solute capacity factor on the column, t_r = compound retention time and t_0 = unretained compound retention time

$$k_{IAM} = \left(\frac{V_s}{V_m}\right) \times K_m$$

where V_s = volume of the IAM interphase created by the immobilized phospholipids, V_m = total volume of the solvent within the IAM column and K_m = membrane partition coefficient

$$V_m = \frac{W_{PhC}}{\delta_{PhC}} + \frac{W_{C10}}{\delta_{C10}} + \frac{W_{C3}}{\delta_{C3}}$$

where the specific weight of PhC (δ_{PhC}) = 1.01779 g/mL and C_{10}/C_3 ($\delta_{C10/C3}$) = 0.86 g/mL; W_{PhC} = 133 mg, W_{C10} = 12.73 mg and W_{C3} = 2.28 mg

$$V_m = f_r \times t_0$$

where f_r = flow rate

$$P_m = \frac{K_m}{MW}$$

where P_m = permeability and MW = molecular weight

Human Serum Albumin (HSA) chromatography for determination of percentage of plasma protein binding (%PPB)

%PPB values were determined using previously developed methodology on a ChromTech HSA 5 µm (3.0 × 50 mm) column. Isopropanol and 0.01 mM phosphate buffered saline at pH 7.4 was used as the mobile phase, with a flow rate of 1.8 mL/min. The retention time of each compound was measured under the following mobile phase conditions: 0–3 min, 0–30% IPA; 3–10 min, 30% IPA; 10.5–11.0 min, 30–0% IPA; 11.0–15.0 min, 0% IPA. System calibration was achieved using the following compounds and plotting %PPB values against their mean retention times: warfarin (%PPB = 98.0), nizatidine (%PPB = 35.0), bromazepam (%PPB = 60.0), carbamazepine (%PPB = 75.0), budesonide (%PPB = 88.0), nicardipine (%PPB = 95.0), ketoprofen (%PPB = 98.7), indomethacin (%PPB = 99.0) and diclofenac (%PPB = 99.8). For each standard compound, the literature %PPB value was converted to its corresponding Log k value, which when plotted against t_r on the HSA column, afforded a line equation from which the Log k value of the unknown compounds could be extracted. The Log k values of the unknown compounds could then be converted to %PPB. Log k and subsequent %PPB calculation for the compounds of interest were performed using Excel 2008 Software.

$$Logk = Log\left[\frac{\%PPB}{(101 - \%PPB)}\right]$$

$$\%PPB = \left[\frac{(101 - 10^{\log k})}{(1 + 10^{\log k})}\right]$$

4. Radioligand Binding Methodology[4]

Preparation of Rat Brain Membranes

Whole brains from male Sprague-Dawley rats (200–300 g) were obtained and immediately added to 25 mL of ice-cold Tris-Base buffer (50 nM, pH 7.4) and thoroughly homogenised using a Polytron. The resultant homogenates were centrifuged at 39100 g for 10 min (4 °C) using a Beckman J2-21M/E centrifuge. After discarding the supernatant, the pellet was resuspended in 25 mL of ice-cold buffer and the centrifugation process repeated. The resultant pellet was then resuspended in 10 mL of ice-cold buffer and the homogenate stored at −50 °C until further use. Total protein content of the brain homogenate was measured using the bicinchoninic acid (BSA) based protein assay. A Bio-Rad solution (solution A/solution B, 50:1) was prepared and 1 mL added to centrifuge tubes. To each tube was added 50 μL of pre-prepared bovine serum albumin (BSA) standards in 50 mM Tris-base buffer (pH 7.4) in the following pre-defined concentrations: 2000, 1500, 1000, 750, 500, 250, 125, 25, and 0 μg/mL. 5 μL of the freshly prepared brain homogenate was added to three centrifuge tubes. The tubes were thoroughly vortexed and incubated at 37 °C for 0.5 h. After incubation, the tubes were left at ambient temperature for 5 min and the absorbance measured at 562 nm using a spectrophotometer. A calibration curve was generated by plotting absorbance against protein content, which was subsequently used to determine the protein content of the rat brain homogenate.

Competition Binding Assays

Brain homogenate stock was diluted to give a final assay concentration of approximately 0.6 mg of protein. Total binding was determined using a final concentration of 1 nM [^{3}H]-PK11195, and non-specific binding was measured using a final concentration of 8 μM unlabelled PK11195 in the presence of [^{3}H]-PK11195. The K_i value of the compound was determined using a range of competitor concentrations (3 pM – 300 μM). Assays were carried out in triplicate, and a typical assay consisted of 100 μL labelled competitor, 100 μL test compound and 200 μL of brain homogenate to give a final assay volume of 400 μL (with <1% ethanol present). The assay components were thoroughly mixed and then incubated at 5 °C for 90 min. The binding assay was terminated by the rapid filtration through Whatman GF/B glass fiber filters, which were pre-soaked in 0.3% w/v polyethylenimine, using a 24-well Brandel cell harvester. Filters received three rapid washes with ice-cold tris-base buffer and were added to prepared scintillation vials containing 10 mL of Eco-scint. After a minimum of 48 h at ambient temperature, tritium counts were measured using a liquid scintillation counter. The resultant radioactive counts were used to determine the K_i

value of the compound of interest by performing non-linear regression analysis using GraphPad Prism 4.0 (GraphPad Softare Inc). Mean and standard deviation (n = 3) was calculated using Excel 2008 software.

5. Binding Curves

PK11195:

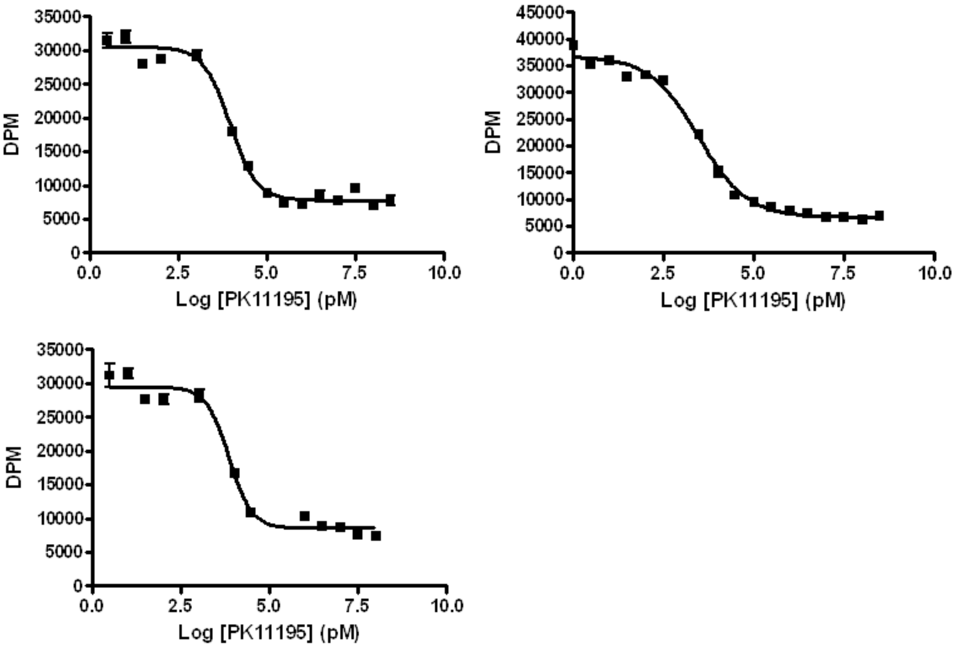

AB5186 6:

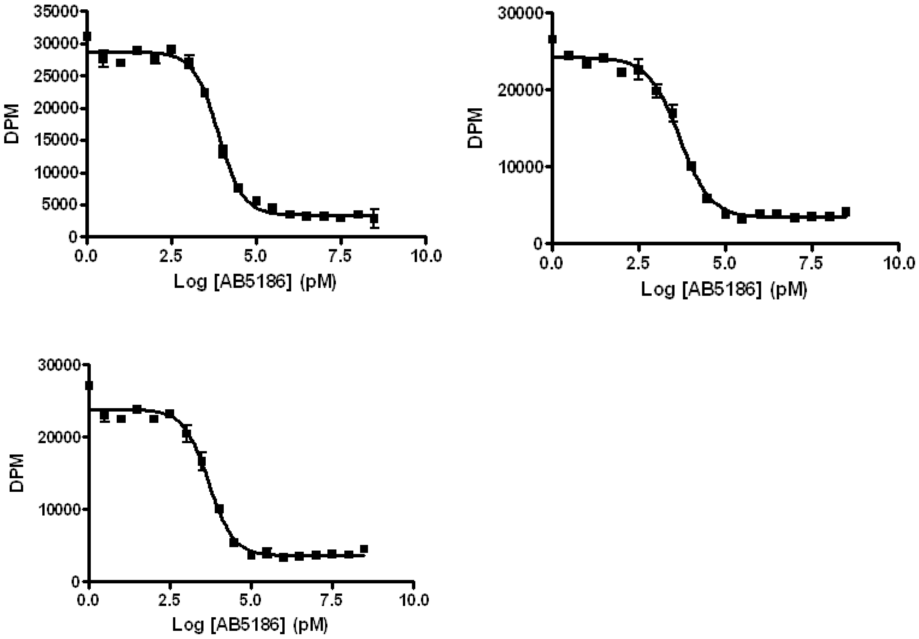

6. Stability Testing

3-(Fluoromethyl)-4-phenylquinoline-2-*N*-diethylcarboxamide (**6**) (2 mg, 0.005 mmol) was dissolved in a 1:1 mixture of 0.01 mM phosphate buffered saline and acetonitrile (2 mL) and incubated at 37 °C. 5 μL of the solution was injected onto the HPLC system (conditions described as below in section 7) at various time points (0, 1, 2, 4, 6 and 24 h). The purity of the compound at these time points was detemined by measuring the area under the peaks of the resultant UV chromatograms.

**Time (h)**	**Purity of Compound (%)**
0	98.81
1	98.75
2	98.76
4	98.80
6	98.78
24	91.94

7. Radiochemistry Methodology

Manual Synthesis of [^{18}F]-AB5186 6

A v-vial containing 0.3 μL of non-carrier added [^{18}F] fluoride (455 MBq) was decapped; to this was added 250 μL of a solution containing 0.106 M Kryptofix® K222 and 0.0347 M potassium carbonate, and then immediately recapped. The fluoride was dried by passing a constant stream of argon over the solution at 100 °C. Additional aliquots of anhydrous acetonitrile (3 × 0.5 mL) was added to facilitate azeotropic drying (approx. 30 min). A solution of the chloride precursor (**13**) (2.4 mg) in acetonitrile (0.5 mL) was then added to the v-vial and the reaction allowed to proceed for 12 mins at 100 °C. After removal from the heating block, the reaction mixture was allowed to cool for a few minutes before being diluted by the addition of 0.21 mL of distilled water.

Semi-Preparative HPLC Purification of [^{18}F]-AB5186 6

The crude ^{18}F-AB5186 reaction mixture was loaded onto a Phenomenex Synergi 4 μm Hydro-RP 80A (150 × 10.00 mm) column. Acetonitrile and water was used as the mobile phase, with a flow rate of 3 mL/min. Purification was achieved using the following isocratic mobile phase: 0 min, 70% acetonitrile; 15 min, 70% acetonitrile; 15.1 min, 95% acetonitrile; 25 min, 95% acetonitrile. The radiolabelled product was detected using a radiodetector, and collected at approximately 8 min.

The fraction containing ^{18}F-AB5186 **6** was concentrated *in vacuo* and then reconstituted in 2 mL of a 10% ethanol/0.9% saline solution. Passing the sample through a pre-treated 13 mm 0.2 μm filter then completed formulation of the radiotracer. The identity of the radiolabelled product was confirmed by co-injection with a cold sample of AB5186 **6**. Quality control was performed by analytical HPLC, using a Phenomenex Synergi 4 μm Hydro-RP 80A (150 × 4.60 mm) column and the mobile phase described above for the semi-prep HPLC purification step. Radiochemical purity was determined by measuring the area under the peaks on the chromatogram, and specific activity was calculated using a standard curve.

8. Radio-HPLC chromatogram of [^{18}F]-AB5186 (6) after purification

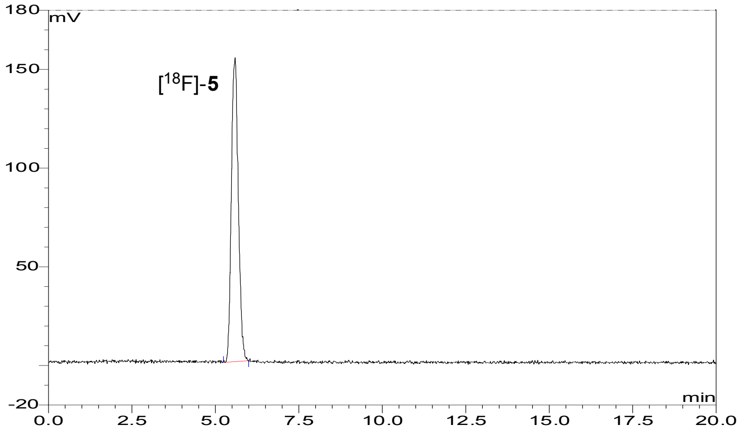

9. Mouse Glioma Models.

CD1 nude mice had 1 × 10^5 of either G7 or U87MG-Luc2 cultured cells implanted intracranially into the caudate nucleus using standard stereotactic procedures. Successful implantation of U87MG-Luc2 tumours was confirmed by performing IVIS® scans approximately two weeks following the procedure. Animals were used for *ex vivo* autoradiography and PET imaging studies 21–30 days post tumour implantation. Prior to any procedure mice were anaesthetised using inhaled isoflurane (in medical oxygen; induction 5.0%; maintenance 2.0–3.0%). Body temperature was maintained using a heating pad.

10. *In vitro* Autoradiography[5] Methodology

The brain from a mouse bearing an intracranial G7 tumour was used. Coronal sections (20 μm thick) were cut in a cryostat at −20 °C from the frozen brain, mounted onto poly-L-lysine coated slides and stored at −50 °C until further use. All incubations and washing steps of the autoradiographic protocol were performed in a freshly prepared 50 mM Tris-base buffer (pH 7.4). Slide mounted brain sections were thawed and pre-washed three times in buffer for 5 min. The slides were then incubated with 3 nM [^{18}F]-AB5186 **6** (7.67 MBq/nmol) for 1 h at ambient

temperature. Non-specific binding was determined in adjacent sections by incubating with [^{18}F]-AB5186 **6** in the presence of 10 μM PK11195 (Tocris, UK). At the end of the incubation, sections were washed twice in ice-cold buffer for 5 min, and then dipped into ice-cold distilled water for 10 s. The slides were subsequently dried at ambient temperature for approximately 10 min by passing a gentle stream of cool air over them. Slides were exposed to [^{3}H]-Hyperfilm (Amersham, UK) for 1 h and then developed. Optical density measurements were made from the resulting autoradiograms using MCID basic 7.0 software (MCID, UK).

11. Immunohistochemistry Methodology

Mice bearing intracranial U87Mg-Luc2 gliomas were perfusion fixed using 4% formaldehyde in PBS. The fixed brains were harvested, embeded in paraffin blocks and 4 μm sections were cut. Paraffin was removed by incubating the tissue sections in xylene (2 × 5 min) followed by a five step rehydration using 100% ethanol (1 min and 5 min), 70% ethanol (2 × 5 min) and water (5 min). Immunohistochemistry was performed using the Dako EnVision™ kits. Briefly, heat induced antigen retrieval using Dako Cytomation Target Retrieval Solution (pH 6.0) in a pressure cooker was followed by an incubation in 3% H_2O_2 in methanol (20 min), and then 5% bovine serum albumin and 5% goat serum in PBS (20 min) to block peroxidase activity and minimise non-specific binding respectively. Between each step the tissue sections were washed in 0.1% Tween® 20 in TBS (2 × 5 min). The brain sections were incubated in either 1:250 anti-TSPO antibody (rabbit anti-human, mouse and rat antibody; NBP1-95674; Novus Biologicals) or 1:250 anti-Iba1 antibody (mouse anti-human, mouse and rat antibody; ab15690; Abcam) overnight at 4–8 ºC. Following this, the tissues were washed with 0.1% Tween® 20 in TBS (2 × 5 min) and a secondary one hour incubation at room temperature was performed using Dako horseradish-peroxidase labelled anti-mouse or anti-rabbit polymers. The sections were once again washed using 0.1% Tween® 20 in TBS (2 × 5 min) and the presence of antibody was detected using the 3,3'-diaminobenzidine chromagen solution diluted in Dako DAB+ substrate buffer. Haematoxylin and eosin staining was performed using a Leica ST5020 multistainer. Histology images were acquired using a Zeiss AX10 brightfield microscope at ×4 magnification and contrast was corrected manually using the ImageJ 1.47v software.

12. *Ex Vivo* Autoradiography Methodology

Mice bearing intracranial U87Mg-Luc2 gliomas received a bolus tail vein injection of either 0.1 mL of vehicle (20% PEG 400 and 5% ethanol in 0.9% saline) (n=4) or 1 mg/kg of PK11195 (Tocris, UK) in 0.1 mL of vehicle (n=4). After 10 min the mice received a slow intravenous injection (0.4 mL/min) of 1.5–2.7 MBq [^{18}F]-AB5186 **6** in 0.2 mL of 10% ethanol in 0.9% saline. After a further

20 min the mice were sacrificed by CO_2 asphyxiation, the brains were removed and frozen using a Cryospray (Cellpath). Mice remained under anaesthesia until the time of cull. The frozen brains were cut at 40 μm using a cryostat at −20 °C (Leica CM1950), transferred to uncoated glass slides (Snowcoat®) and applied to Kodak® BioMax® film. The film was developed following an overnight incubation at −20 °C and scanned using a Bio-Rad GS-800 Calibrated Densitometer with the aid the Quantity One Basic (version 4.6.3) software. Quantification was performed using the ImageJ 1.47v software. A sliding paraboloid background subtraction algorithm was applied with a rolling ball radius of between 30–50 pixels depending on the size of the tumour hotspot. Optical density values were extracted from regions of interest which were drawn manually around the tumour hotspots and mirrored to define the contralateral side. The mean ratios of the tumour to contralateral optical densities were calculated for both the vehicle control and PK11195 (Tocris, UK) blockade cohorts and an unpaired t-test was performed using the GraphPad Prism 6.0 software (GraphPad Softare Inc). Error bars represent the mean SD.

13. Small Animal PET Methodology

Imaging

A single mouse bearing an intracranial U87Mg-Luc2 glioma was anaesthetised and placed in a transaxial position in the Albira imaging system (Carestream Molecular Imaging, USA). The mouse was restrained in the scanner bed and a 120 min dynamic PET scan (1 acquisition/min) was performed following a bolus tail vein injection of 8.2 MBq [^{18}F]-AB5186 **6**. CT was acquired in best-resolution mode (600 projections; exposure time 8 min) using high intensity (400 μA) and low voltage (35 kV) settings. The animal remained anaesthetised during the scan by inhaled isoflurane (in medical oxygen; maintenance 2.5%) and was monitored using a live video feed.

Image Processing and Analysis

The dynamic PET sinograms were corrected for random coincidences, dead time, scatter and decay. Acquired data were reconstructed using the maximum likelihood expectation maximization (MLEM) algorithm with 12 iterations. Data analysis was performed using the PMOD software (PMOD Technologies Ltd. Zurich, Switzerland) version 3.504. The PET image was manually co-registered with the CT and the skull of the animal was used to define the brain. The PET signal outside of the brain region was masked. Frames from the first 10 min of the dynamic PET scan were averaged to allow for tumour hotspot identification. A volume of interest (VOI) was manually drawn around the tumour hotspot region and was used to generate a time-activity curve. Standard uptake values (SUV) were determined as the concentration in the VOI divided by the injected dose

divided by the animal weight. A secondary spherical VOI was used to define the contralateral kinetics and the tumour to contralateral SUV ratio was calculated as a function of time. These data were represented graphically using the GraphPad Prism 6 software (GraphPad Softare Inc).

14. Primate PET Methodology

Imaging

A baboon (*Papio anubis*, 21.3 kg) was fasted for up to 12 h prior to imaging. On the imaging day, the animal was anesthetised with intramuscular ketamine (10.0 mg/kg) and glycopyrrolate (0.1–0.2 mg/kg) at approximately 2 h prior to radiotracer injection, transferred to the Biograph mCT PET/CT scanner (Siemens Healthcare Molecular Imaging, Knoxville, TN, USA), and immediately intubated with an endotracheal tube for continued anesthesia with 1.5–2.5% isoflurane administered through a re-breathing circuit. An intravenous line was established in a cephalic vein for the administration of fluids for hydration and for radiotracer injection. Additionally, an arterial line was established to allow collection of blood samples for metabolite analysis. Body temperature was maintained using a heated water blanket and monitored by rectal thermometer. Vital signs, including end tidal CO_2, SpO_2, heart rate, respiration rate and blood pressure, were monitored continuously and recorded at least every 15 min during the study. The baboon received a bolus intravenous injection of 178.47 MBq [^{18}F]-AB5186 **6** which was immediately followed by a transmission scan and a 240 min emission scan obtained using the three dimensional mode as follows: 6 × 30 s, 3 × 60 s, 2 × 2 min, and 46 × 5 min.

Image Processing and Analysis

The dynamic PET sinograms were corrected for random coincidences, dead time, scatter, decay and attenuation. Acquired data were reconstructed using the ordered subset expectation maximization (OSEM) algorithm. Image analysis was performed using the PMOD software (PMOD Technologies Ltd. Zurich, Switzerland) version 3.405. T1-weighted MR imaging was performed with a Siemens Sonata (1.5T, MPRAGE, repetition time of 2.53 s; echotime of 3.34 ms; inversion time of 1.1 s; and flip angle of 7°). Acquired images were reduced from an initial matrix of 256 × 256 with 176 axial slices (axial slice thickness, 0.6 mm) to a 128 × 128 matrix with 120 axial slices (axial slice thickness, 1.2 mm). Average images of [^{18}F]-AB5168 **6** PET data were generated by averaging the scans with highest brain uptake. Then, the average image was co-registered to the MR image. Finally a volume of interest (VOI) for the whole brain, brainstem, midbrain, hippocampus, parietal cortex, cerebellum, caudate, putamen, globus pallidus, striatum, anterior and posterior cingulate cortex and occipital cortex was applied to the co-registered PET images. Time-activity curves were

generated and SUV determined as the concentration in the VOI divided by the injected dose divided by the animal weight. These data were represented graphically using the GraphPad Prism 6 software (GraphPad Softare Inc).

Primate Blood Analysis

Arterial blood samples were collected from the baboon every 45 s until 6 min after injection of $[^{18}F]$-AB5186 **6** and then at 8, 10, 15, 20, 25, 30, 45, 60, 90, 120, 150, 180 and 240 min. With the exception of the samples collected for metabolite analysis (3.5 mL each), all blood samples were 1 mL each. Samples for metabolite analysis were collected at 6, 15, 30, 60, 120, 150 and 240 min after radiotracer bolus injection. After blood collection, all samples were kept on ice until analysed. Radioactivity in whole blood and plasma was assessed with a well-type γ-counter using a 400–1400 keV window (Perkin Elmer Wallac 2480, USA). Plasma samples were processed by acetonitrile denaturation and analysed by HPLC on a Luna C_{18} column with a mobile phase of $MeOH/H_2O-Et_3N$ (0.2%) 80/20 at a flow rate of 4 mL/min to estimate the parent fraction.

15. Time Activity Curves of [^{18}F]-AB5186 6 in Baboon

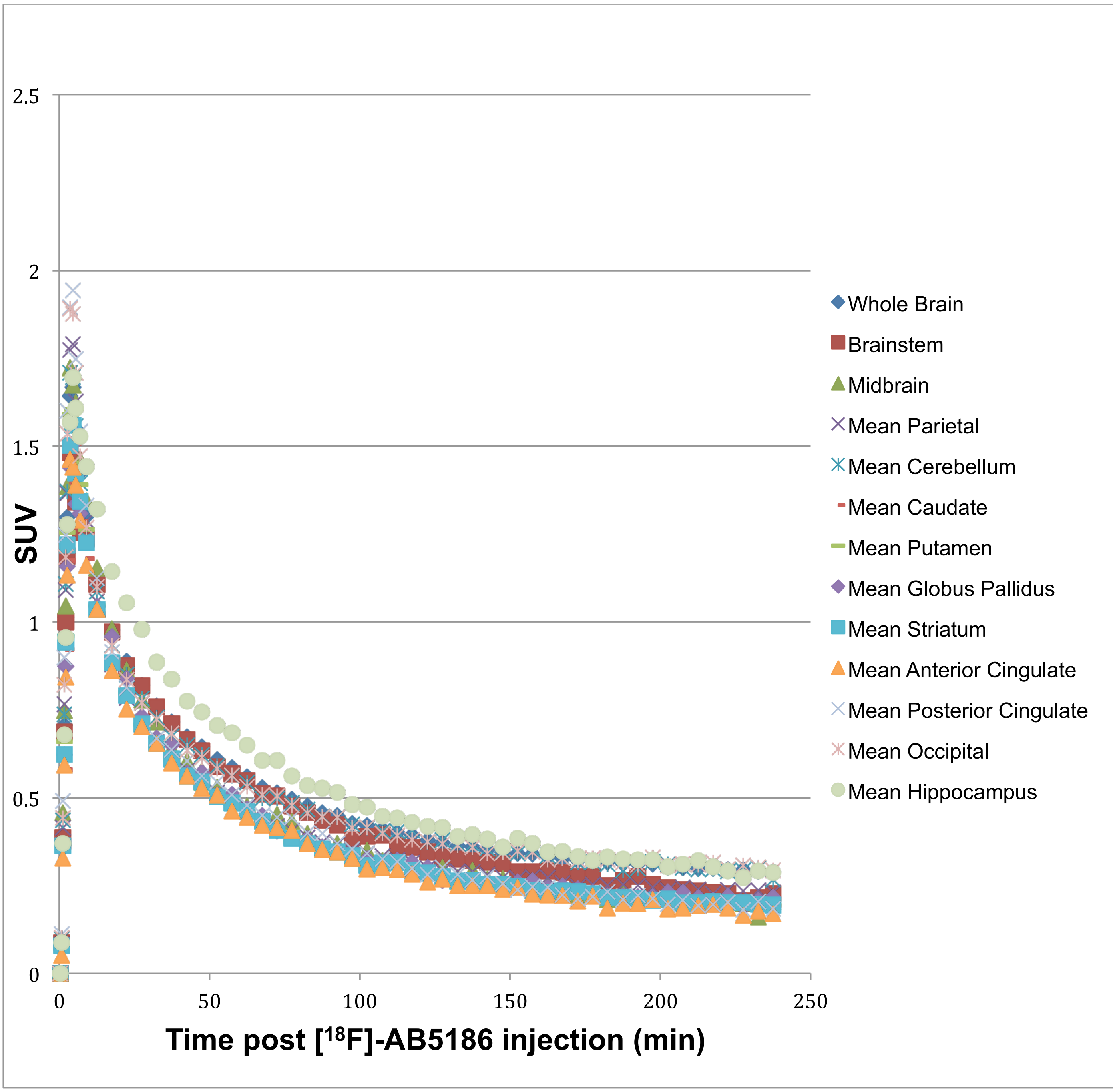

16. References

1. T. Chanda, R. K. Verma and M. S. Singh, *Chem. Asian. J.*, 2012, **7**, 778.

2. M. Anzini, A. Cappelli and S. Vomero, *Heterocycles*, 1994, **38**, 103.

3. A. A. S. Tavares, J. Lewsey, D. Dewar and S. L. Pimlott, *Nucl. Med. Biol.*, 2012, **39**, 127.

4. Y. Itzhak, L. Baker and M. D. Norenberg, *GLIA*, 1993, **9**, 211.

5. D. R. Gehlert, D. T. Stephenson, D. A. Schober, K. Rash and J. A. Clemens, *Neurochem.*, 1997, **31**, 705.

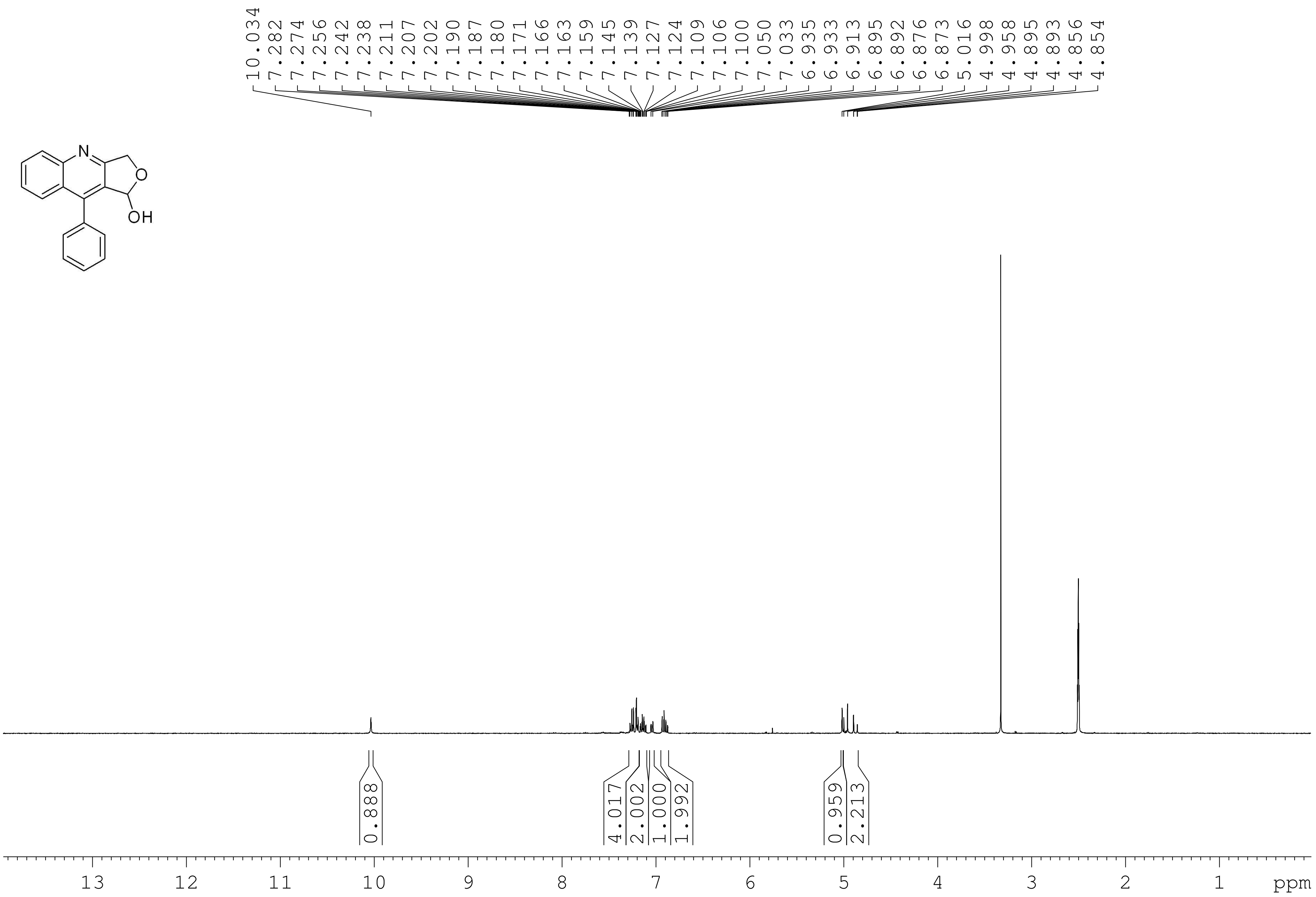
10.034
7.282
7.274
7.256
7.242
7.238
7.211
7.207
7.202
7.190
7.187
7.180
7.171
7.166
7.163
7.159
7.145
7.139
7.127
7.124
7.109
7.106
7.100
7.050
7.033
6.935
6.933
6.913
6.895
6.892
6.876
6.873
5.016
4.998
4.958
4.895
4.893
4.856
4.854
N
O
OH
0.888
4.017
2.002
1.000
1.992
0.959
2.213
13
12
11
10
9
8
7
6
5
4
3
2
1
ppm

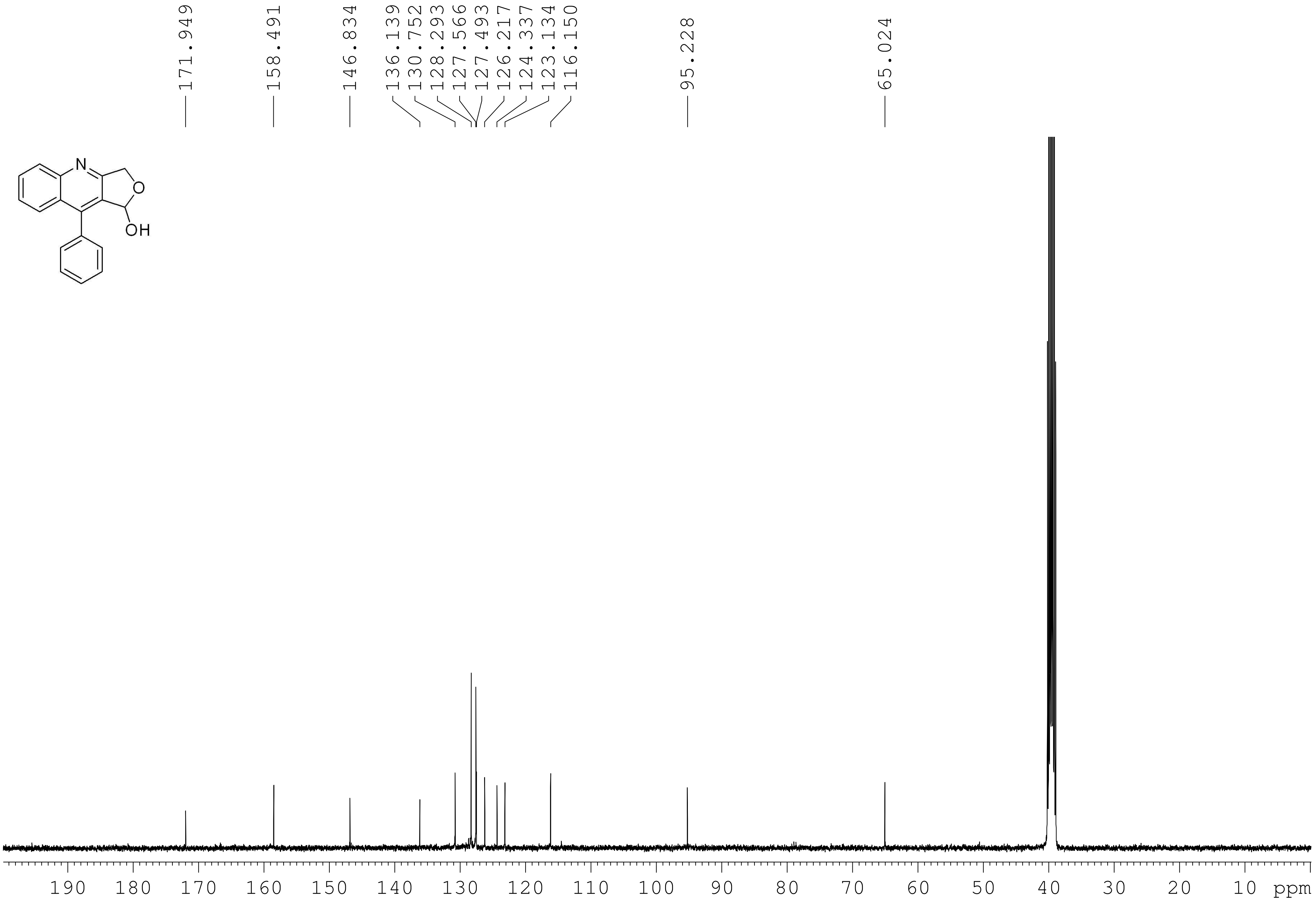
171.949
158.491
146.834
136.139
130.752
128.293
127.566
127.493
126.217
124.337
123.134
116.150
95.228
65.024
N
O
OH
190 180 170 160 150 140 130 120 110 100 90 80 70 60 50 40 30 20 10 ppm

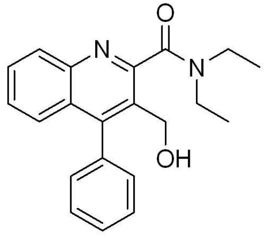

O
N
N
OH

12

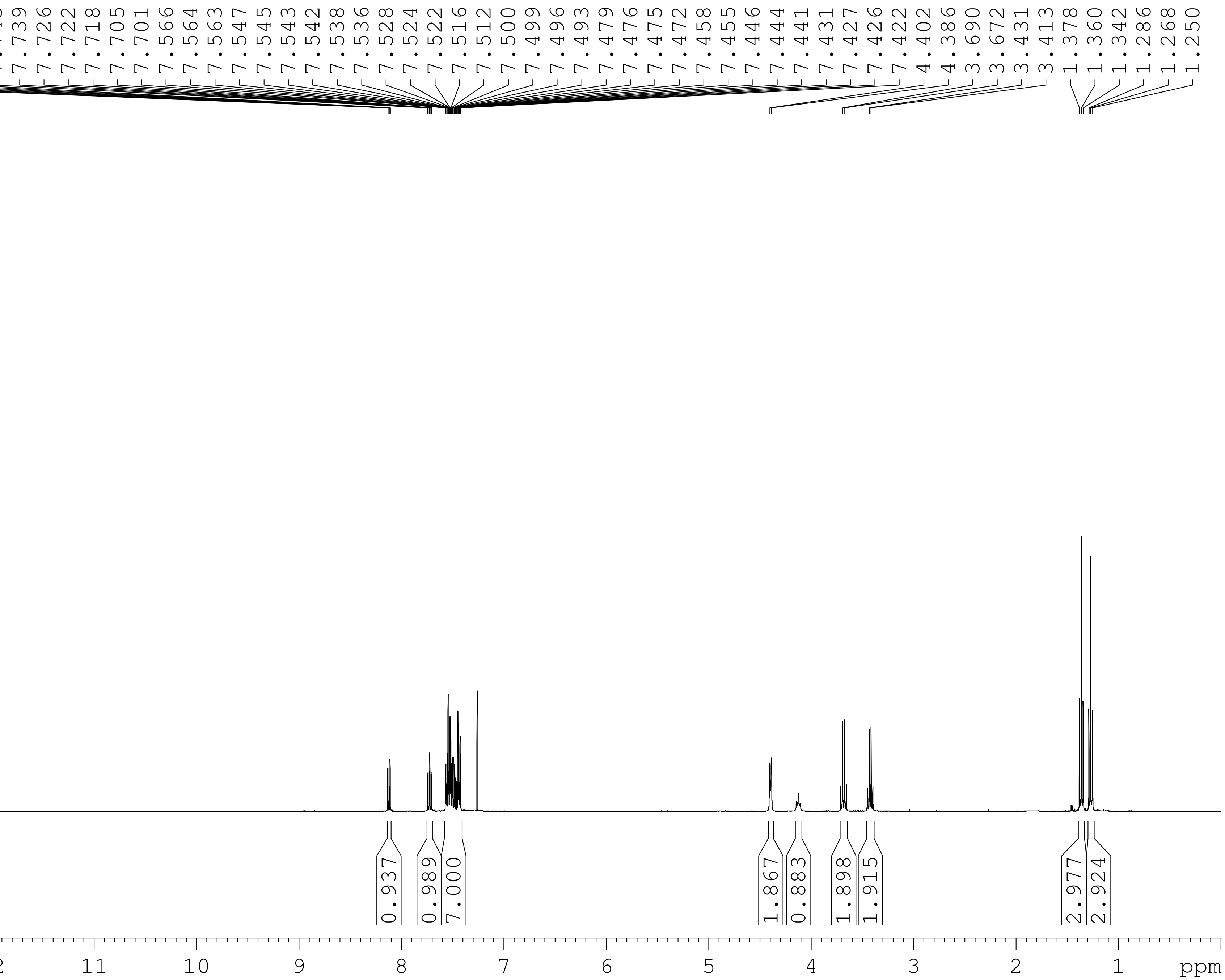

8.131
8.130
8.128
8.112
8.110
8.109
8.107
7.743
7.739
7.726
7.722
7.718
7.705
7.701
7.566
7.564
7.563
7.547
7.545
7.543
7.542
7.538
7.536
7.528
7.524
7.522
7.516
7.512
7.500
7.499
7.496
7.493
7.479
7.476
7.475
7.472
7.458
7.455
7.446
7.444
7.441
7.431
7.427
7.426
7.422
4.402
4.386
3.690
3.672
3.431
3.413
1.378
1.360
1.342
1.286
1.268
1.250
0.937
0.989
7.000
1.867
0.883
1.898
1.915
2.977
2.924
13
12
11
10
9
8
7
6
5
4
3
2
1
ppm

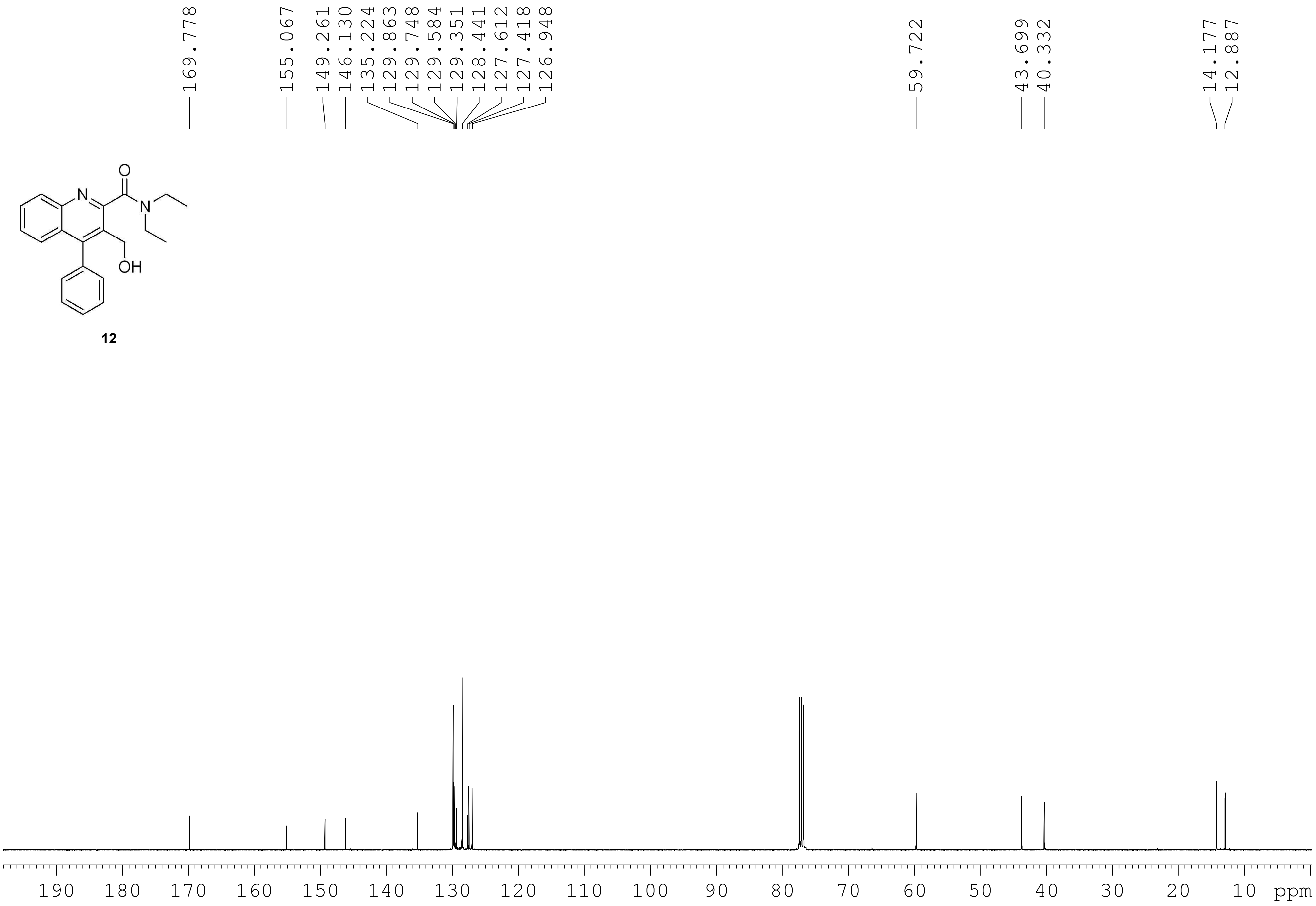
169.778
155.067
149.261
146.130
135.224
129.863
129.748
129.584
129.351
128.441
127.612
127.418
126.948
59.722
43.699
40.332
14.177
12.887
O
N
N
OH
12
190 180 170 160 150 140 130 120 110 100 90 80 70 60 50 40 30 20 10 ppm

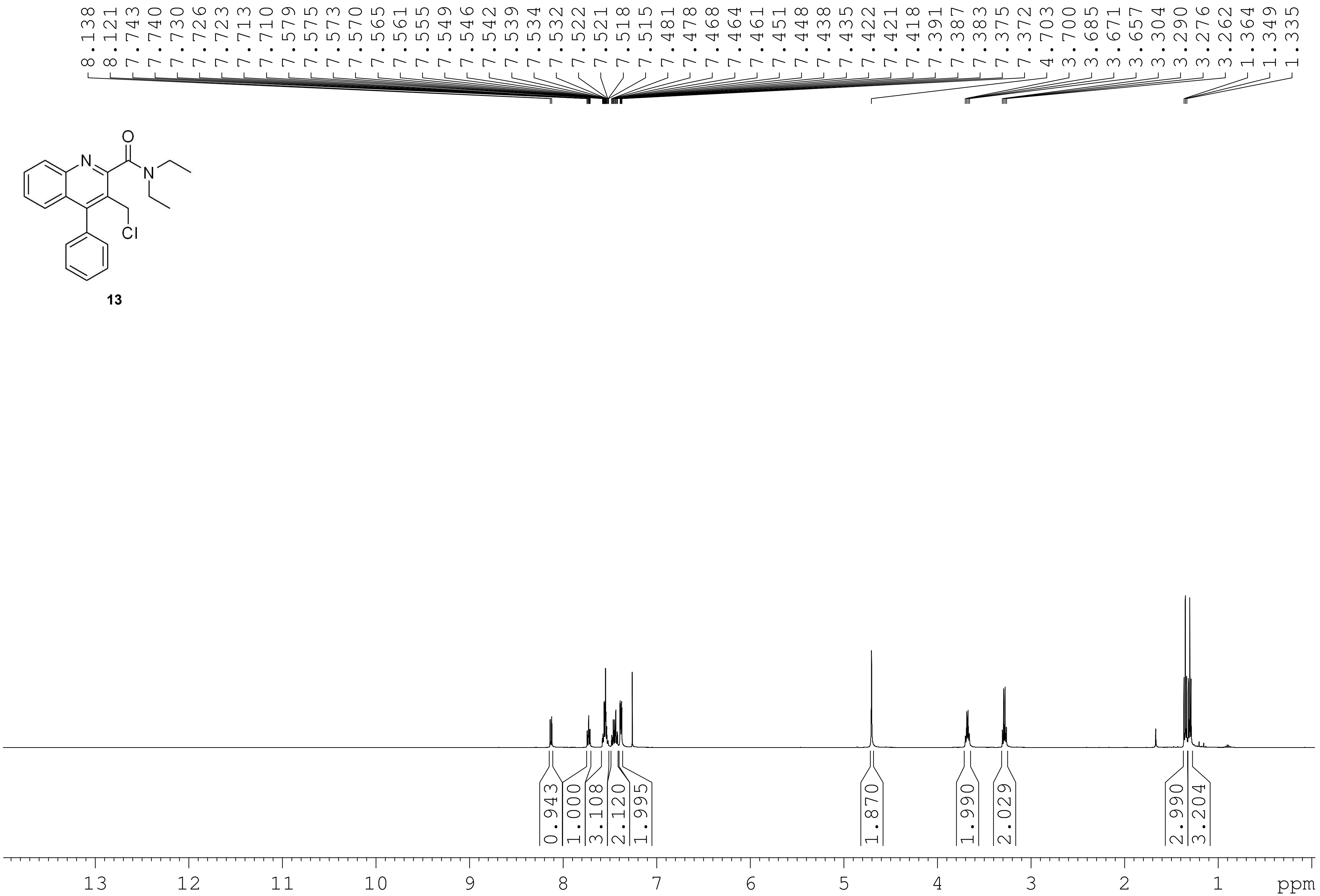
8.138 8.121 7.743 7.740 7.730 7.726 7.723 7.713 7.710 7.579 7.575 7.573 7.570 7.565 7.561 7.555 7.549 7.546 7.542 7.539 7.534 7.532 7.522 7.521 7.518 7.515 7.481 7.478 7.468 7.464 7.461 7.451 7.448 7.438 7.435 7.422 7.421 7.418 7.391 7.387 7.383 7.375 7.372 4.703 3.700 3.685 3.671 3.657 3.304 3.290 3.276 3.262 1.364 1.349 1.335
N
O
N
Cl
13
0.943
1.000
3.108
2.120
1.995
1.870
1.990
2.029
2.990
3.204
13 12 11 10 9 8 7 6 5 4 3 2 1 ppm

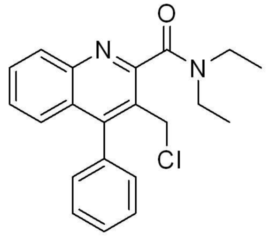
N
O
N
Cl

13

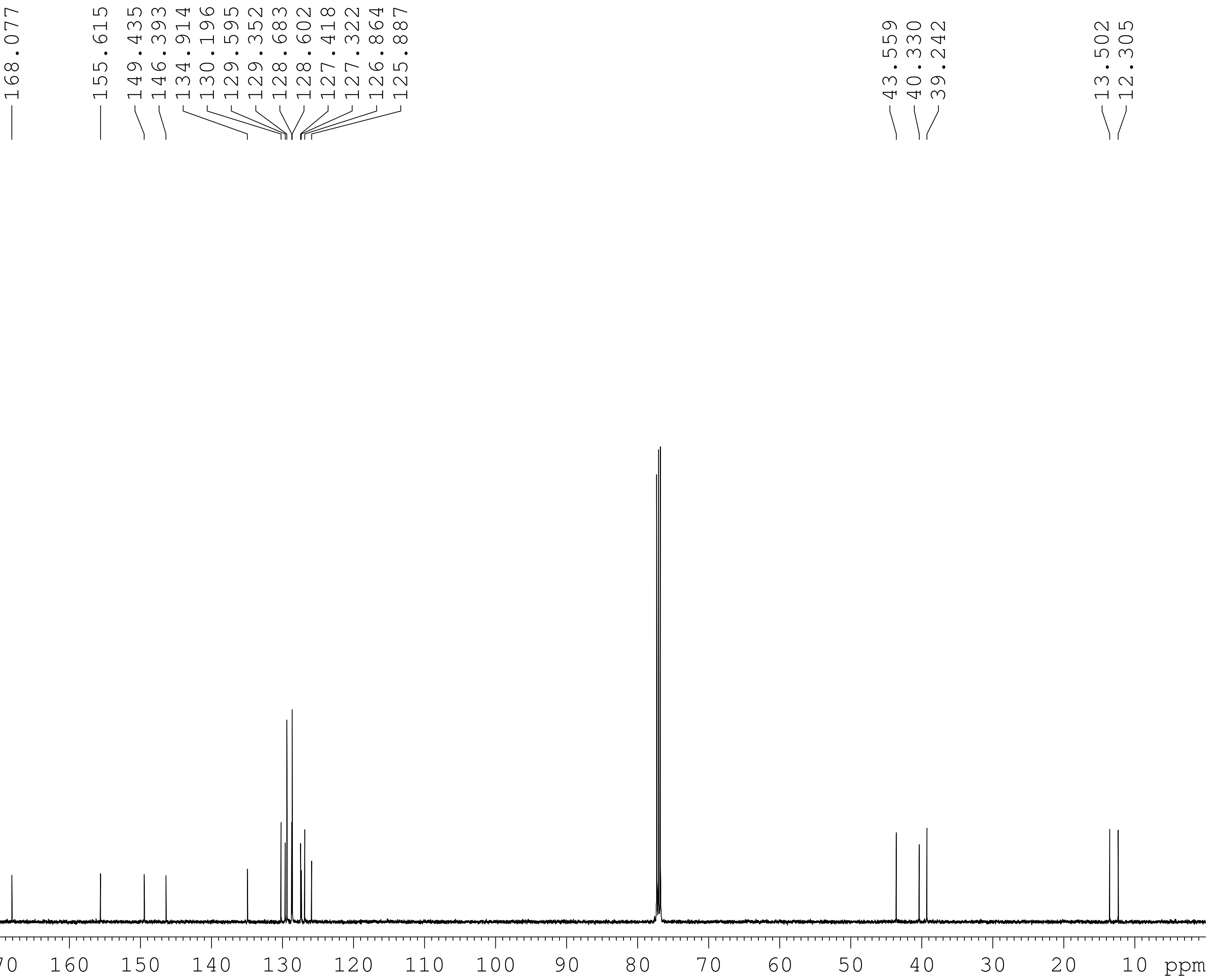
168.077
155.615
149.435
146.393
134.914
130.196
129.595
129.352
128.683
128.602
127.418
127.322
126.864
125.887
43.559
40.330
39.242
13.502
12.305
190 180 170 160 150 140 130 120 110 100 90 80 70 60 50 40 30 20 10 ppm

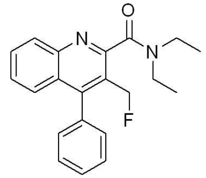

6

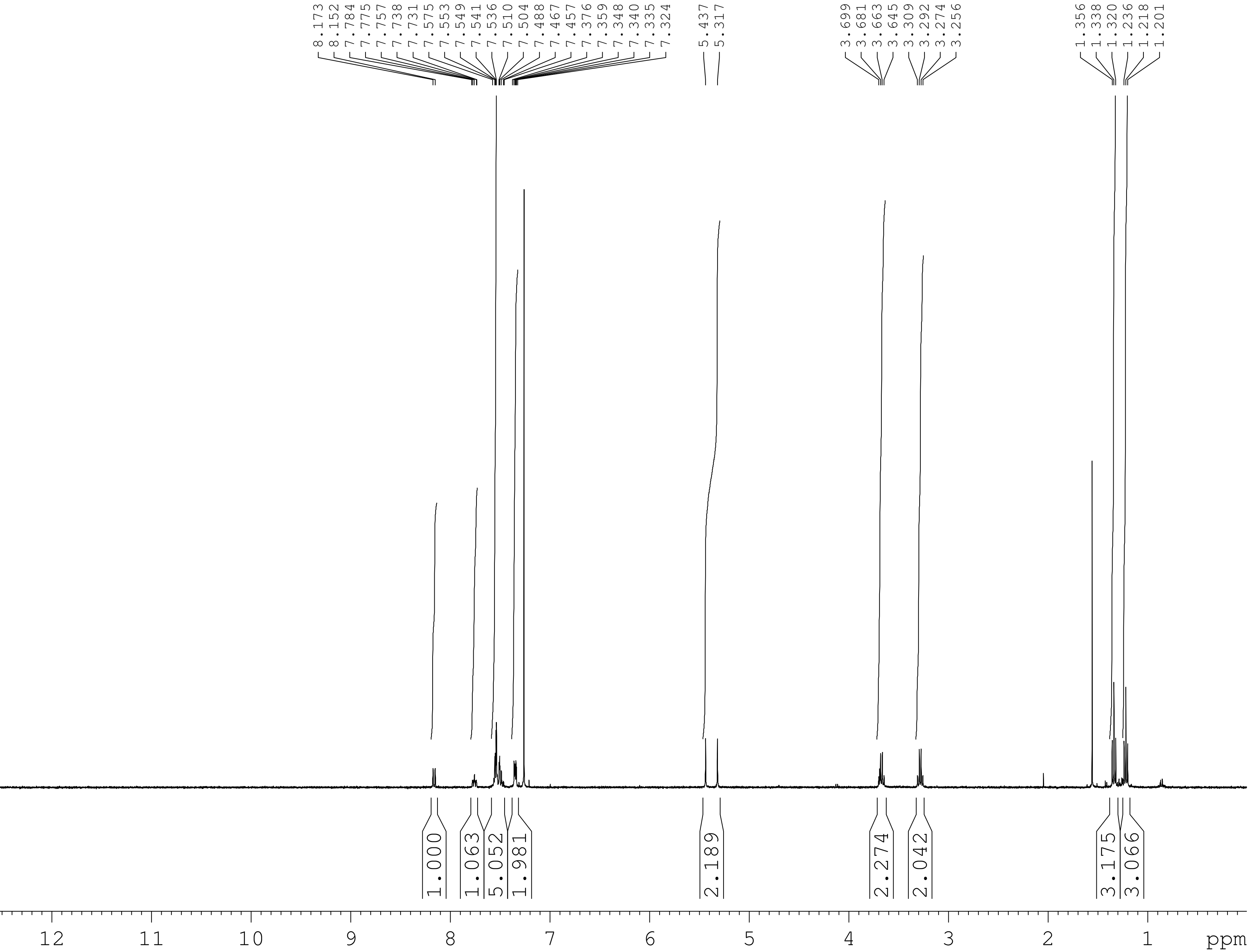
8.173
8.152
7.784
7.775
7.757
7.738
7.731
7.575
7.553
7.549
7.541
7.536
7.510
7.504
7.488
7.467
7.457
7.376
7.359
7.348
7.340
7.335
7.324
5.437
5.317
3.699
3.681
3.663
3.645
3.309
3.292
3.274
3.256
1.356
1.338
1.320
1.236
1.218
1.201
1.000
1.063
5.052
1.981
2.189
2.274
2.042
3.175
3.066
13 12 11 10 9 8 7 6 5 4 3 2 1 ppm

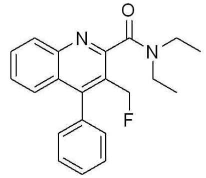

6

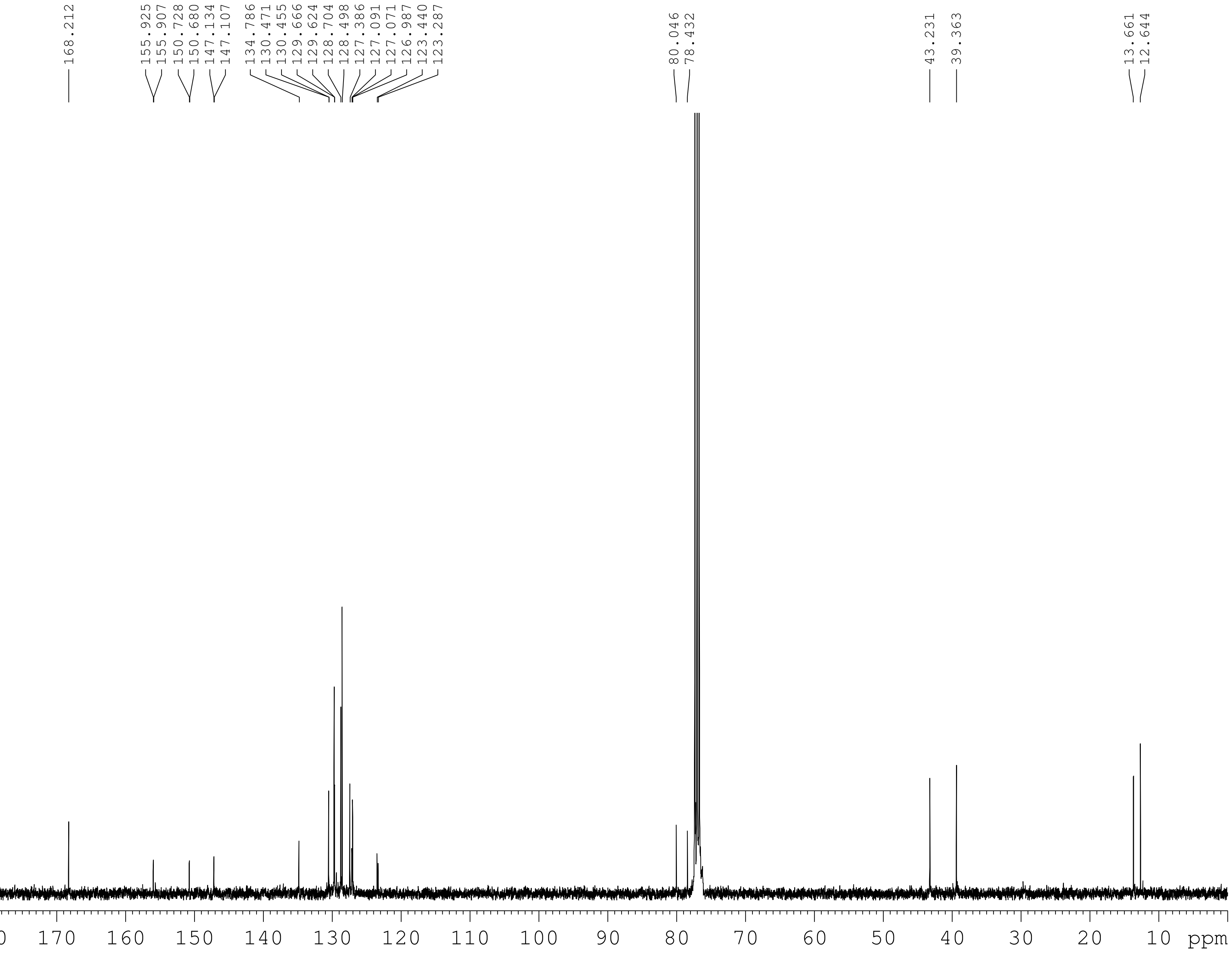

168.212
155.925
155.907
150.728
150.680
147.134
147.107
134.786
130.471
130.455
129.666
129.624
128.704
128.498
127.386
127.091
127.071
126.987
123.440
123.287
80.046
78.432
43.231
39.363
13.661
12.644
190 180 170 160 150 140 130 120 110 100 90 80 70 60 50 40 30 20 10 ppm
